# On the Design of Hyperstable Feedback Controllers for a Class of Parameterized Nonlinearities. Two Application Examples for Controlling Epidemic Models

**DOI:** 10.3390/ijerph16152689

**Published:** 2019-07-27

**Authors:** Manuel De la Sen

**Affiliations:** Institute of Research and Development of Processes IIDP, University of the Basque Country, Campus of Leioa, PO Box 48940 Leioa (Bizkaia), Spain; manuel.delasen@ehu.eus

**Keywords:** Bernouilli, epidemic models, hyperstabily, input-output energy, passivity, positive realness, vaccination controls, treatment controls

## Abstract

This paper studies the hyperstability and the asymptotic hyperstability of a single-input single-output controlled dynamic system whose feed-forward input-output dynamics is nonlinear and eventually time-varying consisting of a linear nominal part, a linear incremental perturbed part and a nonlinear and eventually time-varying one. The nominal linear part is described by a positive real transfer function while the linear perturbation is defined by a stable transfer function. The nonlinear and time-varying disturbance is, in general, unstructured but it is upper-bounded by the combination of three additive absolute terms depending on the input, output and input-output product, respectively. The non-linear time-varying feedback controller is any member belonging to a general class which satisfies an integral Popov’s-type inequality. This problem statement allows the study of the conditions guaranteeing the robust stability properties under a variety of the controllers designed for the controlled system and controller disturbances. In this way, set of robust hyperstability and asymptotic hyperstability of the closed-loop system are given based on the fact that the input-output energy of the feed-forward controlled system is positive and bounded for all time and any given initial conditions and controls satisfying Popov’s inequality. The importance of those hyperstability and asymptotic hyperstability properties rely on the fact that they are related to global closed-loop stability, or respectively, global closed-loop asymptotic stability of the same uncontrolled feed-forward dynamics subject to a great number of controllers under the only condition that that they satisfy such a Popov’s-type inequality. It is well-known the relevance of vaccination and treatment controls for Public Health Management at the levels of prevention and healing. Therefore, two application examples concerning the linearization of known epidemic models and their appropriate vaccination and/or treatment controls on the susceptible and infectious, respectively, are discussed in detail with the main objective in mind of being able of achieving a fast convergence of the state- trajectory solutions to the disease- free equilibrium points under a wide class of control laws under deviations of the equilibrium amounts of such populations.

## 1. Introduction

Studies to design controllers to improve the basic properties of dynamic systems is very important in theoretical studies and in many industrial applications as well as in the study of biological systems and epidemic models under appropriate controls. In particular, all those related models have to be positive in the sense that the solution trajectory cannot take negative values at any time under non-negative initial conditions. For instance, to control the evolution of species in fisheries or to implement feedback vaccination or treatment reasonable techniques on epidemic models which are used to describe infectious diseases affecting to humans, cattle or plants. See, for instance, [1,2,3] and some references therein. It turns out that as models fix better the real dynamics which is tried to describe, more sophisticated tools need to be invoked, like, for instance, nonlinearities of several forms: saturations and dead-zones in physical problems (for instance, the description of combined effects of distinct equilibrium points and the qualitative dynamics of controlled tunnel diodes), quadratic terms, for instance, when describing the infection transmission in epidemic models, or when controlling electrical machinery under jointly non-constant current and voltage. This need of considering nonlinearities overlapped with the basic either linear of linearized dynamics leads to the use of formal absolute stability results for actuator non-linearities belonging to predefined sectors. The existing related background literature is abundant. See, for instance, [4] and references therein. A further step towards a robust controller synthesis is to take into account that the nonlinearities might be also eventually time-varying and that the closed-loop stabilization should be achieved under a certain tolerance to parametrical or structural variations of the controller due, for instance, to ageing of condition changing failures or to construction component dispersion in some relevant parameters. In this context, it has been formulated the hyperstability/asymptotic hyperstability theory which guarantee global stabilization and global asymptotic stabilization for a wide class of controllers provided they satisfy a Popov’s-type integral inequality which is, in fact, a passivity condition of the class of admissible controllers. See, for instance [5,6,7,8,9,10,11,12] as well as some of the references therein. It can also be pointed out that a wide class of existing results on non-linear stability and stabilization synthesis tools, c.f. [13,14,15,16,17,18], can be described in a unified manner under the hyperstablity and passivity theories, [19,20,21], which are more general. See also [22,23,24,25] and references therein for further theoretical and application work of stability, robust stability and hyperstability. It can be pointed out that if a system is described by a positive real transfer functions then its input–output energy defined as the time integral of the input-output product is non-negative under zero initial conditions for any time interval. It can be also pointed out that the theory referred to has not been tried previously for the design of a wide class of non-linear time varying feedback controls for model-based epidemics analysis.

This paper studies the hyperstability and the asymptotic hyperstability of a single-input single-output controlled dynamic systems whose feed-forward input-output dynamics is nonlinear and eventually time-varying consisting of a linear nominal part, a linear incremental perturbed part and a nonlinear and eventually time-varying one subject to a known upper-bounding growing structure. The upper- bounding function consists of weighting additive terms of the absolute values of the measured input, output and input-output product. The nominal linear part is described by a positive real transfer function while the linear perturbation is defined by a stable transfer function. The contribution of the non-nominal part and nonlinear disturbances is compensated by a critical value of the direct input- output interconnection gain. The nonlinear and time-varying disturbance is, in general, unstructured but it is upper-bounded by the combination of three additive absolute terms depending on the input, output and input-output product, respectively. The non-linear time-varying feedback controller is any member belonging to a general class which satisfies an integral Popov’s-type time-integral inequality. The closed-loop system is guaranteed to be either hyperstable or asymptotically hyperstable under the above class on controllers which includes a wised class of members. Section 2 investigates the positivity and the strict positivity for all time of the input-output energy measure of the feed-forward system irrespective of the control law, if any. The obtained conditions are based on either the positive realness or on the strict positive realness of the nominal transfer function provided that the value of the input-output direct interconnection gain exceeds a certain positive threshold. Such a threshold guarantees that such positivity properties are kept under the linear and non-linear uncertainties of such a feed-forward block. Section 3 incorporates non-linear and eventually time-varying control feedback laws which are subject to Popov’s-type inequalities, that is the controllers belong to a passive class. This fact guarantees that the above positivity of the input-output energy of the open-loop system is also finitely uniformly bounded for all time. The final conclusion is that, for the whole number of admissible controllers within the above class, all the relevant variables (that is, controls and state and measured variables) are bounded for all time if the transfer function of the linear part of the feed-forward device is positive real so that the closed-loop system is hyperstable. It has to be pointed out that the hyperstability property refers to a whole class of controllers, not just to an individual one, so that any member of the class is valid to achieve the suitable property. If it is strongly strictly positive real subject to a minimum positive threshold of the direct-input output-interconnection gain then those signals furthermore converge asymptotically to zero as time tends to infinity. Section 4 discusses two examples related to linearized epidemic models around the disease-free equilibrium point subject to feedback vaccination and/or treatment control laws involving feedback information to the light of the above formalism. The so-called incremental vaccination control law satisfies a very general Popov’s-type time-integral inequality. Accordingly to the previous formalism presented and developed in the preceding sections, the vaccination and/ or treatment controllers belong to wide classes of controllers satisfying Popov’s type inequalities in such a way that any member of those classes is useful to achieve the asymptotic stabilization of the incremental dynamics around the disease-free equilibrium point. In this context, the incremental controls and the incremental state and output variables related to the nominal situation converge asymptotically to zero as time tends to infinity. This statement allows to accommodate the implementation of the control laws to the management possibilities like availability, disposal, distribution or costs of the vaccines. Finally, conclusions end the paper. The main contributions of the paper are basically the following ones: (1) to extend the classical hyperstability theory to the presence of unmodeled, or not very precisely parameterized linear and nonlinear contributions to the dynamics, (2) to apply the obtained results to epidemic models under vaccination and treatment incremental controls under a wide class of asymptotically hyperstable controllers, (3) to use those control designs to annihilate the deviations of the steady disease-free equilibrium state due to those kinds of disturbances or unmodeled dynamics and/or changes in the equilibrium population levels. The above concerns have not been previously explored in the existing background literature on the subject. The theoretical application to epidemic models is made basically to the incremental linearized dynamics around the disease- free equilibrium point. This allows the treatability of the formal problem and to take actions against the disease propagation earlier enough when the potential disturbances appear. It is foreseen to extend the results to global asymptotic hyperstability results to the whole nonlinear epidemic models.

### Notation

♦ The superscript T stands for the transposes of vectors and matrices,
♦ $I_{n}$ is the n-th identity matrix,
♦ ∂(N(s)) is the degree of the polynomial N(s),
♦ $i=\sqrt{-1}$ is the complex imaginary unit,
♦ the transfer function G(s) is positive real, denoted by G(s)∈{PR}, if Re G(s)≥0 for all Re s>0,
♦ the transfer function G(s) is strictly positive real, denoted by G(s)∈{SPR}, if for all Re s≥0
♦ the transfer function G(s) is strongly strictly positive real, denoted by G(s)∈{SSPR}, if G(s)∈{SPR} and $\underset{Re\;s\to \infty }{\lim}Re\;G\left(s\right)>0$,
♦ $L_{2}\equiv L_{2}\left[0\;,\infty \right)$, $L_{\infty }\equiv L_{\infty }\left[0,\infty \right)$, C≡C[0,∞), $C^{\left(1\right)}\equiv C^{\left(1\right)}\left[0,\infty \right)$ and PC≡PC[0,∞) are the sets of square-integrable, bounded, continuous, continuous time-differentiable and piece-wise continuous real functions on [0, ∞), respectively. If a superscript n≥2 is added to those sets, say $L_{2}^{n}\equiv L_{2}^{n}\left[0,\infty \right)$, then the involved functions elements are real n-vector functions,
♦ $f\in L_{\infty a}$, $f\in C_{a}$ and $f\in PC_{a}$ if, respectively, $\left(f-f_{0}\right)\in L_{\infty }$, $\left(f-f_{0}\right)\in C$ and $\left(f-f_{0}\right)\in PC$, where $f_{0}:R_{0+}\to R_{0+}$ is some real function of zero support, $RH_{\infty }$ is the set of rational functions with finite $H_{\infty }$ norm. For instance, any strictly stable realizable transfer function belongs to $RH_{\infty }$,
♦ a real n-vector v is nonnegative, referred to as $v\in R_{0+}^{n}$ or as $v\underline{≻}0$, if all its components are non-negative. It is positive, referred to as $v\left(\neq 0\right)\in R_{0+}^{n}$ or as v≻0, if it is non-negative and at least one of its components is positive. It is strictly positive, referred to as $v\in R_{+}^{n}$ or as v≻≻0, if all its components are positive. Note that $v≻≻0\Rightarrow v≻0\Rightarrow v\underline{≻}0$. A real n×m–matrix A is nonnegative, referred to as $A\in R_{0+}^{n\times m}$ or as $A\underline{≻}0$, if all its entries are non-negative. It is positive, referred to as $A\left(\neq 0\right)\in R_{0+}^{n\times m}$ or as A≻0, if it is non-negative and at least one of its entries is positive. It is strictly positive, referred to as $A\in R_{+}^{n\times m}$ or as A≻≻0, if all its entries are positive. Note that $A≻≻0\Rightarrow A≻0\Rightarrow A\underline{≻}0$,
♦ a dynamic system is non-negative (positive) if all the components of their state and output trajectory solutions are non-negative (positive) for all time under non-negative initial conditions and everywhere non-negative controls. A dynamic system is externally non-negative (externally positive) if all the components of its output trajectory solutions are externally non-negative (externally positive) for all time under null initial conditions and everywhere non-negative controls,
♦ $M_{E}^{n\times n}$ is the set of square real Metzler matrices $A=\left(a_{ij}\right)$ of n-th order; i.e., with all their off-diagonal entries being non-negative.

## 2. Positivity of the Input-Output Energy for Unforced and Forced Open-Loop-Controlled Systems

Consider the nonlinear time-varying differential system:

(1) $$\begin{array}{ll} y\left(t\right)=G\left(D\right)u\left(t\right) & +\eta \left(y\left(t\right),\;u\left(t\right)\right)\\ & =\left(G_{0}\left(D\right)+d+\tilde{G}\left(D\right)\right)u\left(y\left(t\right)\right)+\eta \left(y\left(t\right),\;u\left(y\left(t\right)\right)\right) \end{array}$$

where u(t) and y(t) are the input and the output, respectively, D=d/dt is the time-derivative operator, the direct input-output interconnection gain is d≥0 and the rational functions $G_{0}\left(D\right)=\frac{N_{0}\left(D\right)}{M_{0}\left(D\right)}$ and $G\left(D\right)=\frac{N\left(D\right)}{M\left(D\right)}$ have numerator and denominator polynomials $N\left(D\right)=N_{0}\left(D\right)+dM_{0}\left(D\right)$; $M\left(D\right)=M_{0}\left(D\right)$ subject to the degree constraints $\partial M_{0}\left(D\right)>\partial N_{0}\left(D\right)$, $\partial \tilde{M}\left(D\right)>\partial \tilde{N}\left(D\right)$, namely, $G_{0}\left(D\right)$ and $\tilde{G}\left(D\right)$ are strictly proper, and $\partial M\left(D\right)=\partial M_{0}\left(D\right)=\partial N\left(D\right)$ if d>0. Then, $G\left(D\right)=\frac{N\left(D\right)}{D\left(D\right)}=\frac{N_{0}\left(D\right)+dD_{0}\left(D\right)}{D\left(D\right)}$. Note that G(s) and $G_{0}\left(s\right)$ are the transfer functions which model the linear part, where “s” is the Laplace transform argument which is formally identical to the time-derivative operator D=d/dt. In particular, $G_{0}\left(s\right)$ and $G_{1}\left(s\right)$ are the nominal transfer functions excluding and including the direct input-output interconnection gain d and excluding the (non-nominal) linear parametrical contributed disturbances given by $\tilde{G}\left(s\right)$. Note that the direct input-output interconnection gain d is a scalar and $\tilde{G}\left(s\right)$ describes the unmodeled linear dynamics in such a way that the current linear dynamics can be of higher-order than the nominal one. The associated Fourier transforms, referred to as the corresponding frequency responses, if they exist are G(iω) and $G_{0}\left(i\omega \right)$, being got are by taking s=iω in the corresponding transfer functions, where ω=2πf is the angular frequency (given typically, in rad./sec.) and f is the oscillation frequency (given typically in Hertz, that is, cycles per second). A state-space realization of $G_{0}\left(s\right)$ is $R_{0}=\left(A_{0},b_{0},c_{0}^{T}\right)$, such that of $G_{1}\left(s\right)=G\left(s\right)-\tilde{G}\left(D\right)=G_{0}\left(s\right)+d=c_{0}^{T}\left(sI_{n}-A_{0}\right)b_{0}+d$, where $A_{0}\in R^{n\times n}$, $b_{0},c_{0}\in R^{n}$ and d∈R are the system matrix, or matrix of dynamics, control and output matrices, and input-output interconnection gain respectively. A state-space realization of the transfer function $G_{1}\left(s\right)$ in (1) is $R=\left(A_{0},b_{0},c_{0}^{T},\;d\right)$ of state vector $x_{0}\left(t\right)\in R^{n_{0}}$, $n_{0}$ being the order of $A_{0}$ equalizing the degree of $D_{0}\left(s\right)$ and D(s) and also equalizing and number of poles of G(s) excluding possible accounting of zero-pole cancellations, if any. The state and output solution trajectories of the linear system described by G(s) are solution trajectories, under zero initial conditions $D^{i}y_{0}\left(0\right)=0$ for i=0,1,…, n−1 with $D^{0}=1$, of the linear dynamic system of *n*th-order (the number of poles of $G_{0}\left(s\right)$) and state $x_{0}\left(t\right)$:

$${\dot{x}}_{0}\left(t\right)=A_{0}x\left(t\right)+b_{0}u\left(t\right);\;y_{0}\left(t\right)=c_{0}^{T}x\left(t\right)+du\left(t\right)$$

with $x_{0}\left(0\right)=0$ (corresponding to null initial conditions $D^{i}y\left(0\right)=0$ for i=0,1,…,n−1) with $x_{0}:\left[0,\infty \right)\to R^{n}$ and $y_{0}:\left[0,\infty \right)\to R$ being the state and output trajectory solutions for a control input u:[0,∞)→R when the initial conditions are zero which are sometimes referred to as the zero-state and zero-output responses. Thus, the state and output solution trajectories of the linearized nominal Equation (1), associated with the transfer function $G_{1}\left(s\right)$, when $x_{0}\left(0\right)=x_{00}$, $\tilde{G}\left(s\right)=0$ and η≡0 are:

$$\begin{array}{c} x_{0}\left(t\right)=e^{A_{0}t}x_{00}+\int_{0}^{t}e^{A_{0}\left(t-\tau \right)}b_{0}u\left(\tau \right)d\tau ;\;y_{0}\left(t\right)=c_{0}^{T}e^{A_{0}t}x_{00}+\int_{0}^{t}c_{0}^{T}e^{A_{0}\left(t-\tau \right)}b_{0}u\left(\tau \right)d\tau +\\ du\left(t\right);\;\forall t\in R_{0+} \end{array}$$

Similar considerations can be given for a state-space realization of $G_{0}\left(s\right)$. If the contributions of the eventually non-null linear and nonlinear perturbations are included then the state and output trajectory solutions are:

(2) $$x\left(t\right)=e^{At}x_{0}+\int_{0}^{t}e^{A\left(t-\tau \right)}\left[bu\left(\tau \right)+\eta \left(y\left(\tau \right),\;u\left(y\left(\tau \right)\right)\right)\right]d\tau ;\;\forall t\in R_{0+}$$

$$y\left(t\right)-\eta \left(y\left(t\right),\;u\left(y\left(t\right)\right)\right)=c^{T}e^{At}x_{0}+\int_{0}^{t}c^{T}e^{A\left(t-\tau \right)}bu\left(\tau \right)d\tau +du\left(t\right);\;\forall t\in R_{0+}$$

with $x\left(0\right)=x_{0}$, where $x\left(t\right)\in R^{n}$, $n>n_{0}$ being the total added number of poles of G(s) and $\tilde{G}\left(s\right)$ without excluding the accounting of potential zero-pole cancellations, if any, and R=(A,b,c,d) is a state-space realization of $G\left(s\right)+\tilde{G}\left(s\right)=G_{0}\left(s\right)+\tilde{G}\left(s\right)+d$. If the system (1) is controlled under a nonlinear output- feedback control then the nonlinear time-varying control law will be assumed in Section 3 to be of class $\Phi_{0*}$:

(3) u(t)=u(y(t),t)=−ϕ(y(t),t)

where $\phi \in \Phi_{0*}$, with $\Phi_{0*}=\cup_{\gamma \in R_{0+}}\Phi_{0\gamma }$ being the class of hyperstable controllers, where

(4) $$\Phi_{0\gamma }=\left\{\phi :R_{0+}\times R_{0+}\to R\;|\int_{0}^{t}\phi \left(y\left(\tau \right),\tau \right)y\left(\tau \right)d\tau \geq -\gamma >-\infty ;\forall t\in R_{0+}\right\};\;\gamma \in R_{0+}$$

Note that if $\phi \in \Phi_{0*}$ (that is, $\phi \in \Phi_{0\gamma }$ for some $\gamma \in R_{0+}$) then $\underset{t\to \infty }{\lim\;inf}\int_{0}^{t}\phi \left(y\left(\tau \right),\tau \right)y\left(\tau \right)d\tau >-\infty$. The time-integral defining the set $\Phi_{0\gamma }$ is known as Popov’s inequality. Let the Fourier transforms of y(t) and u(t) be $Y\left(i\omega \right)=F\left(y\left(t\right)\right)=\int_{-\infty }^{\infty }y\left(t\right)e^{-i\omega t}dt$ and U(iω)=F(u(t)) with $i=\sqrt{-1}$ is the complex imaginary unit. Let f: R→R. Therefore, if the input is absolutely integrable then the zero state output is absolutely integrable as well since $G\left(s\right)=G_{0}\left(s\right)+d$ is stable since $G_{0}\left(s\right)\in \left\{PR\right\}$ implies that G(s)∈{PR} where “s” is the Laplace transform argument. Note that for the existence of the Fourier transform of a real function it suffices that it be absolutely integrable on R. The truncation of f(τ) in the interval [0,t] is defined for any $t\in R_{0+}$ as $f_{t}\left(\tau \right)=f\left(\tau \right)$ for τ∈[0,t] and $f_{t}\left(\tau \right)=0$ for τ∈(−∞,0)∪(0, ∞). As a result of the sufficiency of the absolute integrability for the existence of Fourier transforms, the Fourier transform $F_{t}\left(i\omega \right)$ of the truncated function $f_{t}\left(\tau \right)$ on [0,t] always exist for any finite t since $\int_{0}^{t}\left|f\left(\tau \right)\right|d\tau =\int_{-\infty }^{\infty }\left|f_{t}\left(\tau \right)\right|d\tau$. Thus, the Fourier transforms of the input and output on any finite time interval always exist since those of the corresponding truncated functions always exist. The input-output energy in the interval ${\left[t_{1},t_{2}\right]}^{}$ is defined as $E\left(t_{1},t_{2}\right)=\int_{t_{1}}^{t_{2}}y\left(\tau \right)u\left(\tau \right)d\tau$.

*Assumptions*

**A1.** $\left|\eta \left(t\right)\right|\leq k_{1}\left|u\left(t\right)\right|+k_{2}\left|y\left(t\right)\right|+k_{3}\left|y\left(t\right)u\left(t\right)\right|$ *for some* $k_{i}\in R_{+}$ (i=1,2,3)
**A2.** $G_{0}\left(s\right)\in \left\{PR\right\}$ *and* $\underset{\omega \in R_{0+}}{sup}\left|\tilde{G}\left(i\omega \right)\right|\leq M_{\tilde{G}}<\infty$
**A3.** $\infty >d>\underset{\omega \in R_{0+}}{max}\left|\tilde{G}\left(i\omega \right)\right|+k_{1}-\underset{\omega \in R_{0+}}{min}Re\;G_{0}\left(i\omega \right)\geq 0$
**A4.**$\underset{0\leq \tau \leq t}{sup}\left|u\left(\tau \right)\right|\leq \frac{1-k_{2}}{k_{3}}$*;*$\forall t\in R_{0+}$*if*$k_{3}\neq 0$*and*$k_{2}<1$.

The following result relies on the positivity on any positive time interval of non-zero measure of the input-output energy $E\left(0,t\right)=\int_{0}^{t}y\left(\tau \right)u\left(\tau \right)d\tau$ of the controlled or uncontrolled system on the time interval [0, t] under the above assumptions. It is independent of the system being in open-loop (i.e., uncontrolled with u≡0) or of the used controller if the controlled system is subject to feedback. Its proof is based on expressing equivalently the input-output energy in the frequency domain for any finite time-interval by using Parseval’s theorem for the Fourier transform of the truncated input and output on such a finite interval.

**Theorem** **1.** *If Assumptions A1–A4 hold then:*

$$\begin{array}{l} E\left(0,t\right)\geq \frac{\left(d+\underset{\omega \in R_{0+}}{min}Re\;G_{0}\left(i\omega \right)-\underset{\omega \in R_{0+}}{max}\left|Re\;\tilde{G}\left(i\omega \right)\right|-k_{1}\right)\int_{0}^{t}u^{2}\left(\tau \right)d\tau }{\left(1+k_{2}+k_{3}\underset{0\leq \tau \leq t}{sup}\left|u\left(\tau \right)\right|\right)}>\\ \;>0 \end{array}$$

*for any given*$t\in R_{+}$*under zero initial conditions if*u(t)*is nonzero on some time interval of finite measure of*[0,t]*. If*u≡0*, or if, more generally, it has a support of zero measure, then*E(0,t)≥0*;*$\forall t\in R_{0+}$.

*In particular, if*η≡0*, then:*

$$E\left(0,t\right)\geq \left(d+\underset{\omega \in R_{0+}}{min}Re\;G_{0}\left(i\omega \right)-\underset{\omega \in R_{0+}}{max}\left|Re\;\tilde{G}\left(i\omega \right)\right|\right)\int_{0}^{t}u^{2}\left(\tau \right)d\tau >0$$

*under zero initial conditions if*u(t)*is nonzero on some time interval of finite measure of*[0,t]*for a given*t>0.

**Proof.**  The input-output energy under zero initial conditions, i.e., the zero-sate input output- energy, that is that associated with zero initial conditions $x_{0}=0$ of (1)) is:

(5) $$\begin{array}{l} E\left(0,t\right)=E_{z.s.}\left(0,t\right)=\int_{0}^{t}y\left(\tau \right)u\left(\tau \right)d\tau \\ \;=\int_{0}^{t}\left(G_{0}\left(D\right)+d+\tilde{G}\left(D\right)\right)u^{2}\left(\tau \right)d\tau +\int_{0}^{t}\eta \left(y\left(\tau \right),\;u\left(y\left(\tau \right)\right)\right)u\left(\tau \right)d\tau \\ \;=\int_{-\infty }^{\infty }y_{t}\left(\tau \right)u_{t}\left(\tau \right)d\tau =\int_{-\infty }^{\infty }y_{t}\left(\tau \right)u\left(\tau \right)d\tau =\int_{-\infty }^{\infty }y\left(\tau \right)u_{t}\left(\tau \right)d\tau \\ \;=\frac{1}{2\pi }\int_{-\infty }^{\infty }\left(G_{0}\left(i\omega \right)+d+\tilde{G}\left(i\omega \right)\right)U_{t}\left(i\omega \right)U_{t}\left(-i\omega \right)d\omega \\ \;+\int_{0}^{t}\eta \left(y\left(\tau \right),\;u\left(y\left(\tau \right)\right)\right)u\left(\tau \right)d\tau \end{array}$$

after using the truncated functions and Parseval’s theorem. From Assumptions A1 and A2 and, since the hodograph $G_{0}\left(i\omega \right)$ has the symmetry and anti-symmetry properties: $Re\;G_{0}\left(i\omega \right)=Re\;G_{0}\left(-i\omega \right)$, $Re\;\tilde{G}\left(i\omega \right)=Re\;\tilde{G}\left(-i\omega \right)$, $Im\;G_{0}\left(i\omega \right)=-Im\;G_{0}\left(-i\omega \right)$ and $Im\;\tilde{G}\left(i\omega \right)=Im\;\tilde{G}\left(-i\omega \right)$; ∀ω∈R, one gets:

(6) $$\begin{array}{l} E\left(0,t\right)\\ \geq |\frac{1}{2\pi }\left|\int_{-\infty }^{\infty }\left(G_{0}\left(i\omega \right)+d\right)U_{t}\left(i\omega \right)U_{t}\left(-i\omega \right)d\omega \right|-|\frac{1}{2\pi }\int_{-\infty }^{\infty }\tilde{G}\left(i\omega \right)U_{t}\left(i\omega \right)U_{t}\left(-i\omega \right)d\omega \\ +k_{1}\int_{0}^{t}u^{2}\left(\tau \right)d\tau +k_{2}\left|\int_{0}^{t}u\left(\tau \right)y\left(\tau \right)d\tau \right|+k_{3}|\int_{0}^{t}y\left(\tau \right)u^{2}\left(\tau \right)d\tau ||\\ \geq |\frac{1}{2\pi }|\int_{-\infty }^{\infty }(d\\ +\underset{\omega \in R_{0+}}{min}Re\;G_{0}\left(i\omega \right))U_{t}\left(i\omega \right)U_{t}\left(-i\omega \right)d\omega |-|\frac{1}{2\pi }\int_{-\infty }^{\infty }\tilde{G}\left(i\omega \right)U_{t}\left(i\omega \right)U_{t}\left(-i\omega \right)d\omega \\ +k_{1}\int_{0}^{t}u^{2}\left(\tau \right)d\tau +k_{2}\left|\int_{0}^{t}u\left(\tau \right)y\left(\tau \right)d\tau \right|+k_{3}|\int_{0}^{t}y\left(\tau \right)u^{2}\left(\tau \right)d\tau ||\\ \geq \left(d+\underset{\omega \in R_{0+}}{min}Re\;G_{0}\left(i\omega \right)-\underset{\omega \in R_{0+}}{max}\left|Re\;\tilde{G}\left(i\omega \right)\right|-k_{1}\right)\int_{0}^{t}u^{2}\left(\tau \right)d\tau -k_{2}\left|\int_{0}^{t}u\left(\tau \right)y\left(\tau \right)d\tau \right|\\ -k_{3}|\int_{0}^{t}y\left(\tau \right)u\left(\tau \right)d\tau |\underset{0\leq \tau \leq t}{sup}\left|u\left(\tau \right)\right|\\ =\left(d+\underset{\omega \in R_{0+}}{min}Re\;G_{0}\left(i\omega \right)-\underset{\omega \in R_{0+}}{max}\left|Re\;\tilde{G}\left(i\omega \right)\right|-k_{1}\right)\int_{0}^{t}u^{2}\left(\tau \right)d\tau \\ -\left(k_{2}+k_{3}\underset{0\leq \tau \leq t}{sup}\left|u\left(\tau \right)\right|\right)\left|E\left(0,t\right)\right| \end{array}$$

since $\underset{\omega \in R_{0+}}{min}Re\;G_{0}\left(i\omega \right)\geq 0$ from Assumption A2. Then, consider two cases for each $t\in R_{0+}$, namely,

Case a: E(0,t)=|E(0,t)|≥0 for a given $t\in R_{+}$ under any nonzero initial conditions. Then:

(7) $$\begin{array}{l} \left(1+k_{2}+k_{3}\underset{0\leq \tau \leq t}{sup}\left|u\left(\tau \right)\right|\right)E\left(0,t\right)\\ \;\geq \left(d+\underset{\omega \in R_{0+}}{min}Re\;G_{0}\left(i\omega \right)-\underset{\omega \in R_{0+}}{max}\left|Re\;\tilde{G}\left(i\omega \right)\right|-k_{1}\right)\int_{0}^{t}u^{2}\left(\tau \right)d\tau \end{array}$$

with E(0,0)=0. Thus, by Assumption A3, E(0,t)>0 for such a $t\in R_{+}$.

Case b: E(0,t)=−|E(0,t)|<0 for a given $t\in R_{+}$ if u(t) is everywhere non-negative on $R_{0+}$ and non-zero on some time interval of finite measure of [0,t]. Then, instead of (7), one gets from (6):

$$\begin{array}{c} -\left|E\left(0,t\right)\right|\geq \left(d+\underset{\omega \in R_{0+}}{min}Re\;G_{0}\left(i\omega \right)-\underset{\omega \in R_{0+}}{max}\left|\tilde{G}\left(i\omega \right)\right|-k_{1}\right)\int_{0}^{t}u^{2}\left(\tau \right)d\tau \\ -\left(k_{2}+k_{3}\underset{0\leq \tau \leq t}{sup}\left|u\left(\tau \right)\right|\right)\left|E\left(0,t\right)\right| \end{array}$$

so that:

(8) $$\begin{array}{l} \left(k_{2}+k_{3}\underset{0\leq \tau \leq t}{sup}\left|u\left(\tau \right)\right|-1\right)\left|E\left(0,t\right)\right|\\ \;\geq \left(d+\underset{\omega \in R_{0+}}{min}Re\;G_{0}\left(i\omega \right)-\underset{\omega \in R_{0+}}{max}\left|\tilde{G}\left(i\omega \right)\right|-k_{1}\right)\int_{0}^{t}u^{2}\left(\tau \right)d\tau >0 \end{array}$$

if u(t) is everywhere non-negative on $R_{0+}$ and non-zero on some time interval of finite measure of [0,t], Thus, from Assumptions A3 and A4, since |E(0,t)|≥0 for all $t\in R_{0+}$, the left-hand- side of (8) is negative so that Equation (8) yields the contradiction 0 < 0. Thus Case b is impossible and E(0,t)>0, subject to (7), for all $t\in R_{+}$. On the other hand, if η≡0 then the corresponding particular part of the proof follows by taking $k_{1}=k_{2}=k_{3}=0$. The proof is complete. □

**Remark** **1.** *Note that if*$G_{0}\left(s\right)\in \left\{PR\right\}$*then it is analytical in*Re s≥0*, i.e., stable, that is, with all its poles in*Re s≤0*and of relative degree (i.e., the pole-zero excess number)*+1,0,−1*and any critically stable pole of*G(s)*(i.e., such that*Re s=0*), if any, is single and with associated positive residue. If*$G_{0}\left(s\right)\in \left\{SPR\right\}$*then it is analytical in*Re s>0*, i.e., strictly stable, that is, with all its poles in*Re s<0.

**Remark** **2.** *If*$G_{0}\left(s\right)\in \left\{PR\right\}$*and*$G_{0}^{-1}\left(s\right)$*are both strictly stable (i.e.,*$G_{0}\left(s\right)$*is positive real, it has all its poles and zeros in*Re s<0*) and Assumption A3 holds with sufficiently large*d>0*then*G(s)∈{SSPR}, and it has zero relative degree.

Since a transfer function which is strongly strictly positive real is also strictly positive real and then positive real as a result, the following corollary to Theorem 1 is obvious since if $G_{0}\left(s\right)\in \left\{SPR\right\}$ then it is strictly stable:

**Corollary** **1.** *Theorem 1 holds, in particular, if Assumptions A1, A3 and A4 hold and, furthermore, the subsequent assumption also holds:*

**A5.**$G_{0}\left(s\right)\in \left\{SPR\right\}$*and*$\tilde{G}\left(s\right)$*is strictly stable with*$\underset{\omega \in R_{0+}}{sup}\left|\tilde{G}\left(i\omega \right)\right|\leq M_{\tilde{G}}<\infty$.

**Remark** **3.** *Note that Assumption A5 is guaranteed under Assumption A6 below:*

**A6.**$G_{0}\left(s\right)\in \left\{SSPR\right\}$*and*$\tilde{G}\left(s\right)$*is strictly stable with*$\underset{\omega \in R_{0+}}{sup}\left|\tilde{G}\left(i\omega \right)\right|\leq M_{\tilde{G}}<\infty$.

**Remark** **4.** *Note that if* $G_{0}\left(s\right)\notin \left\{PR\right\}$ *but it is still stable, i.e., all its poles are in* Re s≤0 *, then Theorem 1 can be extended under a modified Assumption A2 and a more restrictive modified Assumption A3 with a larger value of the input-output interconnection gain d as follows:*

*Assumptions*

**A2’.** $G_{0}\left(s\right)$ *and* $\tilde{G}\left(s\right)$ *are stable and they have no poles at the origin*
**A3’.** $\infty >d>k_{1}+\underset{\omega \in R_{0+}}{max}\left|Re\;\left(G_{0}\left(i\omega \right)+\tilde{G}\left(i\omega \right)\right)\right|\geq 0$

**Theorem** **2.** *If Assumptions A1, A2’, A3’ and A4 hold then:*

$$E\left(0,t\right)\geq \frac{\left(d-\underset{\omega \in R_{0+}}{max}\left|Re\;\left(G_{0}\left(i\omega \right)+\tilde{G}\left(i\omega \right)\right)\right|-k_{1}\right)\int_{0}^{t}u^{2}\left(\tau \right)d\tau }{\left(1+k_{2}+k_{3}\underset{0\leq \tau \leq t}{sup}\left|u\left(\tau \right)\right|\right)}>0$$

*for any given*t>0*under zero initial conditions if*u(t)*is nonzero on some time interval of finite measure of*[0,t]*. If*u≡0*for all time then*E(0,t)≥0*;*$\forall t\in R_{0+}$.

**Outline** **of** **Proof.** It is similar to that of Theorem 1 with the proposed changes in the assumptions which lead to direct modifications in the appropriate equations of the proof by noting that

$$\left(1-k_{2}-k_{3}\underset{0\leq \tau \leq t}{sup}\left|u\left(\tau \right)\right|\right)E\left(0,t\right)\geq \left(d+\underset{\omega \in R_{0+}}{min}Re\;G_{0}\left(i\omega \right)-\underset{\omega \in R_{0+}}{max}\left|Re\tilde{G}\left(i\omega \right)\right|-k_{1}\right)\int_{0}^{t}u^{2}\left(\tau \right)d\tau .$$

 □

**Remark** **5.** *Note that Assumption A3 requires that* $\left|\tilde{G}\left(i\omega \right)\right|$ *be small enough and, in particular, its resonance peak has to satisfy the constraints:*

$$\max\left(\underset{\omega \in R_{0+}}{max}\left|Re\;\tilde{G}\left(i\omega \right)\right|,\underset{\omega \in R_{0+}}{min}Re\;G_{0}\left(i\omega \right)-k_{1}\right)\leq \underset{\omega \in R_{0+}}{max}\left|\tilde{G}\left(i\omega \right)\right|<d+\underset{\omega \in R_{0+}}{min}Re\;G_{0}\left(i\omega \right)-k_{1}$$

**Remark** **6.** *Note that if* $G_{0}\left(s\right)\in \left\{SPR\right\}$ *then Assumption A2 holds, i.e.,* $G_{0}\left(s\right)\in \left\{PR\right\}$ *and Theorem 1 still holds under Assumptions A1–A4 with this more restrictive condition of Assumption A2*

Note that Theorem 1 establishes that the zero-state input-output energy on a nonzero finite time interval [0,t] is positive under Assumption A1–A4 if the input is nonzero on some subinterval of nonzero measure. The following result relies on weakening Assumption A4 if the input converges to zero and it is bounded on [0,∞) concluding in the non-negativity of the input-output energy on any time interval $\left[t_{1},t_{2}\right]$ of nonzero measure.

**Theorem** **3.** *If Assumptions A1–A3 hold with*$k_{2}<1$*,*$u:R_{0+}\to R$*is everywhere bounded on its definition domain, i.e.,*$u\in L_{\infty }$*, and*u(t)→0*as*t→∞*then:*

$$E_{z.s.}\left(t_{1},t\right)\geq \frac{\left(d+\underset{\omega \in R_{0+}}{min}Re\;G_{0}\left(i\omega \right)-\underset{\omega \in R_{0+}}{max}\left|Re\;\tilde{G}\left(i\omega \right)\right|-k_{1}\right)\int_{0}^{t}u^{2}\left(\tau \right)d\tau }{\left(1+k_{2}+k_{3}\underset{0\leq \tau \leq t}{sup}\left|u\left(\tau \right)\right|\right)}\geq 0$$

*for any*$t>t_{1}$*for any given*$t_{1}\in R_{0+}$*. The result holds also if Assumptions A1–A3 hold with*$k_{2}<1$*and*$u\in PC\cap L_{2}$.

**Proof.**  Assume that the initial conditions are neglected. Then, the zero-state input-output energy on the time interval of nonzero measure $\left[t_{1},\;t\right]$ for any given $t_{1}\in R_{0+}$ is after generalizing (5):

(9) $$\begin{array}{l} E_{z.s.}\left(t_{1},t\right)=\int_{t_{1}}^{t}y\left(\tau \right)u\left(\tau \right)d\tau =\int_{-\infty }^{\infty }y\left(\tau \right)u_{\left[t_{1},t\right)}\left(\tau \right)d\tau \\ \;=\int_{t_{1}}^{t}\left(G_{0}\left(D\right)+d+\tilde{G}\left(D\right)\right)u^{2}\left(\tau \right)d\tau +\int_{t_{1}}^{t}\eta \left(y\left(\tau \right),\;u\left(y\left(\tau \right)\right)\right)u\left(\tau \right)d\tau \\ \;=\frac{1}{2\pi }\int_{-\infty }^{\infty }\left(G_{0}\left(i\omega \right)+d+\tilde{G}\left(i\omega \right)\right)U_{\left[t_{1},t\right)}\left(i\omega \right)U_{\left[t_{1},t\right)}\left(-i\omega \right)d\omega \\ \;+\int_{t_{1}}^{t}\eta \left(y\left(\tau \right),\;u\left(y\left(\tau \right)\right)\right)u\left(\tau \right)d\tau \end{array}$$

where the truncation $f_{\left[t_{1},t\right)}=f\left(t\right)$; $\forall t\in \left[t_{1},\;t\right]$ and f(t)=0 for $t\notin \left[t_{1},t\right]$ and any given $t_{1}\in R_{0+}$ is used. Since $k_{2}<1$ and $u:R_{0+}\to R$ is bounded with u(t)→0 as t→∞ then there exists a finite $t^{*}\in R_{0+}$ such that $\underset{t^{*}\leq \tau <\infty }{sup}\left|u\left(\tau \right)\right|<\frac{1-k_{2}}{k_{3}}$. Then one gets that:

(10) $$\begin{array}{l} \left(k_{2}+k_{3}\underset{t^{*}\leq \tau \leq t}{sup}\left|u\left(\tau \right)\right|-1\right)\left|E\left(0,t\right)\right|\\ \;\geq \left(d+\underset{\omega \in R_{0+}}{min}Re\;G_{0}\left(i\omega \right)-\underset{\omega \in R_{0+}}{max}\left|Re\;\tilde{G}\left(i\omega \right)\right|-k_{1}\right)\int_{t*}^{t}u^{2}\left(\tau \right)d\tau >0 \end{array}$$

cannot hold for any $t\in \left[t^{*},\infty \right)$ (see Case b in the proof of Theorem 1), and then:

(11) $$\begin{array}{l} \left(1+k_{2}+k_{3}\underset{t^{*}\leq \tau \leq t}{sup}\left|u\left(\tau \right)\right|\right)E\left(0,t\right)\\ \;\geq \left(d+\underset{\omega \in R_{0+}}{min}Re\;G_{0}\left(i\omega \right)-\underset{\omega \in R_{0+}}{max}\left|Re\;\tilde{G}\left(i\omega \right)\right|-k_{1}\right)\int_{t*}^{t}u^{2}\left(\tau \right)d\tau \end{array}$$

(see Case a in the proof of Theorem 1). On the other hand, note that if $u\in PC\cap L_{2}$ then $u\in L_{\infty }$ and u(t)→0 as t→∞ and the result still holds. □

The subsequent results establish that if the linear part of the system is externally positive then the positivity of the input-output energy through time is kept even under any non-negative initial conditions.

**Corollary** **2.** *If Assumptions A1–A4 hold and, in addition, a state-space realization*R=(A,b,c,d)*of*$G\left(s\right)+\tilde{G}\left(s\right)$*is externally positive (or, alternatively, a state-space realization of*$G\left(s\right)+\tilde{G}\left(s\right)-d$*is externally positive and*d≥0*) and*$\eta :R_{0+}\times R_{0+}\times \left[0,t\right)\to R_{0+}$*, then*E(0,t)≥0*;*$\forall t\in R_{+}$*provided that*$x_{0}\underline{≻}0$*and*u(t)≥0*;*$\forall t\in R_{0+}$.

**Proof.**  It follows that the input-output energy E(0,t) on [0,t] consists of its jointly unforced contribution $E_{u}\left(0,t\right)$ and its forced contribution $E_{f}\left(0,t\right)$ corresponding to zero initial conditions:

(12) $$E\left(0,t\right)=\int_{0}^{t}y\left(\tau \right)u\left(\tau \right)d\tau =E_{u}\left(0,t\right)+E_{f}\left(0,t\right);\;\forall t\in R_{0+}$$

where:

(13) $$E_{u}\left(0,t\right)=c^{T}\int_{0}^{t}e^{A\tau }x_{0}u\left(\tau \right)d\tau ;\;\forall t\in R_{0+}$$

(14) $$E_{f}\left(0,\;t\right)=c^{T}\int_{0}^{t}\int_{0}^{\tau }e^{A\left(\tau -\sigma \right)}\left[bu\left(\sigma \right)+\eta \left(y\left(\sigma \right),\;u\left(y\left(\sigma \right)\right)\right)\right]u\left(\sigma \right)d\sigma d\tau +d\int_{0}^{t}u^{2}\left(\tau \right)d\tau ;\;\forall t\in R_{0+}$$

 □

Note that $E_{u}\left(0,t\right)\geq 0$ and $E_{f}\left(0,t\right)\geq 0$, $\forall t\in R_{0+}$ from Theorem 1for any nonzero input on some subinterval of [0,t] of nonzero measure since $\eta :R_{0+}\times R_{0+}\times \left[0,t\right)\to R_{0+}$ and the state-space realization R=(A,b,c,d) of the transfer function $G\left(s\right)+\tilde{G}\left(s\right)$ is externally positive what implies that $c^{T}e^{At}b+d≻0$; $\forall t\in R_{0+}$.

**Corollary** **3.** *If Assumptions A1, A2’, A3’and A4 hold and, in addition, a state-space realization*R=(A,b,c,d)*of*$G\left(s\right)+\tilde{G}\left(s\right)$*is externally positive (or, alternatively, a state-space realization of*$G\left(s\right)+\tilde{G}\left(s\right)-d$*is externally positive and*d≥0*) and*$\eta :R_{0+}\times R_{0+}\times \left[0,t\right)\to R_{0+}$*then the system is externally positive then*E(0,t)≥0*;*$\forall t\in R_{+}$*under any initial output conditions*$x\left(0\right)=x_{0}\underline{≻}0$*provided that*u(t)≥0*;*$\forall t\in R_{0+}$.

**Remark** **7.** *Note that Assumption A2’ is satisfied in particular if* $G_{0}\left(s\right)$ *and* $\tilde{G}\left(s\right)$ *are stable with no poles at the origin which guarantees that* $\left|Re\;\left(G_{0}\left(i\omega \right)+\tilde{G}\left(i\omega \right)\right)\right|$ *is finite and* $\underset{\omega \in R_{0+}}{sup}\left|\tilde{G}\left(i\omega \right)\right|\leq M_{\tilde{G}}<\infty$ *. However, the key constraint to ensure the input-output energy positivity is not the stability of the transfer functions but the absence of poles at the origin of those transfer functions. The following theorem states results on boundedness of the relevant signals and global stability of the open-loop system; i.e., in the case when the control is not generated via the feedback of the form (3) and (4):*

**Theorem** **4.** *The following properties hold:*

***(i)****Assume that*$\underset{0\leq t<\infty }{sup\left|u\left(t\right)\right|}\leq m_{u}<\frac{1-k_{2}}{k_{3}}$*and that*G(s)*and*$\tilde{G}\left(s\right)$*are stable transfer functions. If*$u:R_{0+}\to R$*is, furthermore, square-integrable, satisfying*$\int_{0}^{\infty }u^{2}\left(\tau \right)d\tau \leq M_{u}^{2}<\infty$*, then*y(t)*is uniformly bounded on*$R_{0+}$*for any given initial conditions according to:*

$$\left|y\left(t\right)\right|\leq \frac{1}{1-k_{2}-k_{3}m_{u}}\times \left(\sqrt{K_{0}}\left(\sqrt{K_{0}}⥂\left|c_{0}^{T}x_{00}\right|+\left|c_{0}^{T}b_{0}\right|M_{u}\right)+\sqrt{\tilde{K}}(\sqrt{\tilde{K}}\left|{\tilde{c}}^{T}{\tilde{x}}_{00}\right|+\;\left|{\tilde{c}}^{T}\tilde{b}\right|M_{u})+\left(k_{1}+d\right)m_{u}\right);\forall t\in R_{0+}$$

*where*$R=\left(c^{T},A,b,d\right)$*,*$R_{0}=\left(c_{0}^{T},A_{0},b_{0},d\right)$*and*$\tilde{R}=\left({\tilde{c}}^{T},\tilde{A},\tilde{b}\right)$*are state space realizations of*G(s)*,*$G_{0}\left(s\right)$*and*$\tilde{G}\left(s\right)$*, respectively, and*$\|e^{A_{0}t}\|\leq K_{0}e^{-\rho_{0}t}=K_{0}$*and*$\|e^{\tilde{A}t}\|\leq \tilde{K}e^{-\tilde{\rho }t}=\tilde{K}$*for some real constants*$K_{0}\geq 1$*,*$\tilde{K}\geq 1$*,*$\rho_{0}=\tilde{\rho }=0$*and;*$\forall t\in R_{0+}$*and*$x_{00}=x_{0}\left(0\right)\in R^{n_{0}}$*and*${\tilde{x}}_{00}={\tilde{x}}_{0}\left(0\right)\in R^{{\tilde{n}}_{0}}$*are the respective initial states of*R*and*$R_{0}$.

***(ii)*** *Assume that* $\underset{0\leq t<\infty }{sup\left|u\left(t\right)\right|}\leq m_{u}<\frac{1-k_{2}}{k_{3}}$ *and that* G(s) *and* $\tilde{G}\left(s\right)$ *are strictly stable transfer functions. Then,* y(t) *is uniformly bounded on* $R_{0+}$ *for any given finite initial conditions according to:*

$$\left|y\left(t\right)\right|\leq \frac{1}{1-k_{2}-k_{3}m_{u}}\left(\left|c_{0}^{T}e^{A_{0}t}x_{00}\right|+\left|{\tilde{c}}^{T}e^{\tilde{A}t}{\tilde{x}}_{00}\right|+\left(\left|c_{0}^{T}b_{0}\right|\frac{K_{0}}{\rho_{0}}+\left|{\tilde{c}}^{T}\tilde{b}\right|\frac{\tilde{K}}{\tilde{\rho }}+\left(k_{1}+d\right)\right)m_{u}\right)\;\leq \frac{1}{1-k_{2}-k_{3}m_{u}}\left(K_{0}\left(\left|c_{0}^{T}x_{00}\right|+\left|c_{0}^{T}b_{0}\right|\frac{m_{u}}{\rho_{0}}\right)+\tilde{K}\left(\left|{\tilde{c}}^{T}{\tilde{x}}_{00}\right|+\left|{\tilde{c}}^{T}\tilde{b}\right|\frac{m_{u}}{\tilde{\rho }}\right)+\left(k_{1}+d\right)m_{u}\right);\;\forall t\in R_{0+}\;\underset{t\to \infty \left|y\left(t\right)\right|\left(\left|c_{0}^{T}b_{0}\right|\frac{K_{0}}{\rho_{0}}+\left|{\tilde{c}}^{T}\tilde{b}\right|\frac{\tilde{K}}{\tilde{\rho }}+\left(k_{1}+d\right)\right)\frac{m_{u}}{1-k_{2}-k_{3}m_{u}}}{lim\;sup}$$

***(iii)*** y(t) *is uniformly bounded on* $R_{0+}$ *for any given finite initial conditions if* $d=k_{1}=k_{3}=0$ *and* $k_{2}<1$ *for any control* $u\in L_{2}$ *if* $G\left(s\right)=G_{0}\left(s\right)+d$ *and* $\tilde{G}\left(s\right)$ *are strictly stable transfer functions satisfying:*

$$\left|y\left(t\right)\right|\leq \frac{1}{1-k_{2}}\left(\left|c_{0}^{T}e^{A_{0}t}x_{00}\right|+\left|{\tilde{c}}^{T}e^{\tilde{A}t}{\tilde{x}}_{00}\right|+\left(\left|c_{0}^{T}b_{0}\right|\frac{K_{0}}{\rho_{0}}+\left|{\tilde{c}}^{T}\tilde{b}\right|\frac{\tilde{K}}{\tilde{\rho }}\right)M_{u}\right)\;\leq \frac{1}{1-k_{2}}\left(K_{0}\left(\left|c_{0}^{T}x_{00}\right|+\left|c_{0}^{T}b_{0}\right|\frac{M_{u}}{\rho_{0}}\right)+\tilde{K}\left(\left|{\tilde{c}}^{T}{\tilde{x}}_{00}\right|+\left|{\tilde{c}}^{T}\tilde{b}\right|\frac{M_{u}}{\tilde{\rho }}\right)\right);\;\forall t\in R_{0+}\;\underset{t\to \infty \left|y\left(t\right)\right|\left(\left|c_{0}^{T}b_{0}\right|\frac{K_{0}}{\rho_{0}}+\left|{\tilde{c}}^{T}\tilde{b}\right|\frac{\tilde{K}}{\tilde{\rho }}\right)\frac{M_{u}}{1-k_{2}}}{lim\;sup}$$

*If the input is jointly uniformly bounded and square-integrable with* $\underset{0\leq t<\infty }{sup\left|u\left(t\right)\right|}\leq m_{u}$ *and* $\int_{0}^{\infty }u^{2}\left(\tau \right)d\tau \leq M_{u}^{2}<+\infty$ *then an asymptotic absolute output upper-bound being irrespective of the initial conditions is:*

$$\underset{t\to \infty \left|y\left(t\right)\right|\left(\left|c_{0}^{T}b_{0}\right|\frac{K_{0}}{\rho_{0}}+\left|{\tilde{c}}^{T}\tilde{b}\right|\frac{\tilde{K}}{\tilde{\rho }}\right)\frac{{\bar{M}}_{u}}{1-k_{2}}}{lim\;sup}$$

*with* ${\bar{M}}_{u}=max\left(m_{u},\;M_{u}\right)$ *. If, in addition,* $k_{3}\neq 0$ *and provided that* ${\bar{M}}_{u}<\frac{1-k_{2}}{k_{3}}$ *then*

$$\underset{t\to \infty \left|y\left(t\right)\right|\left(\left|c_{0}^{T}b_{0}\right|\frac{K_{0}}{\rho_{0}}+\left|{\tilde{c}}^{T}\tilde{b}\right|\frac{\tilde{K}}{\tilde{\rho }}\right)\frac{{\bar{M}}_{u}}{1-k_{2}-k_{3}{\bar{M}}_{u}}}{lim\;sup}$$

***(iv)****If Assumption A1 holds,*$k_{2}<1$*and*$u\in L_{2}$*then*$\left|\left|y\right|-\left|\eta \right|\right|,y,\eta ,\left|y-\eta \right|\in L_{2}$*for any finite initial conditions. If, furthermore,*$G_{0}\left(s\right)$*and*$\tilde{G}\left(s\right)$*are strictly stable then*$x\in L_{2}^{n}$.

***(v)****If Assumption A1 holds,*$k_{2}<1$*and*$u\in L_{2}$*and*$\underset{t\geq t_{a}}{sup}\left|u\left(t\right)\right|<+\infty$*for some finite*$t_{a}\in R_{0+}$*then*$\left|\left|y\right|-\left|\eta \right|\right|,y,\eta ,\left|y-\eta \right|,\|x\|\in L_{2}$*for any finite initial conditions and their supreme are bound on a connected time interval of infinite measure. Explicitly, one has:*

$$\|y_{\infty }\|\leq \frac{m_{u}\left({\left\|\bar{G}\right\|}_{\infty }+k_{1}\right)}{1-k_{2}-k_{3}m_{u}}+K_{1};\;\|y_{2}\|\leq \frac{\left({\left\|\bar{G}\right\|}_{\infty }+k_{1}\right)}{1-k_{2}-k_{3}m_{u}}M_{u}+K_{2}$$

*for some*$0\leq K_{i}=K_{i}\left(x_{0}\right)<+\infty$*;*i=1,2*, where*$\bar{G}\left(s\right)=G\left(s\right)+\tilde{G}\left(s\right)$.

**Proof.**  One gets from (1) that:

(15) $$\begin{array}{l} \left|y\left(t\right)\right|=\left|c^{T}e^{At}x_{0}\right|+\left|c^{T}\int_{0}^{t}e^{A\left(t-\tau \right)}bu\left(\tau \right)d\tau \right|+\left|\eta \left(t\right)\right|\\ \;\leq \left|c^{T}e^{At}x_{0}\right|+\left|c^{T}\int_{0}^{t}e^{A\left(t-\tau \right)}bu\left(\tau \right)d\tau \right|+k_{1}\left|u\left(t\right)\right|+k_{2}\left|y\left(t\right)\right|\\ \;+k_{3}\left|y\left(t\right)u\left(t\right)\right|\\ \;\leq \left|c_{0}^{T}e^{A_{0}t}x_{00}\right|+\left|{\tilde{c}}^{T}e^{\tilde{A}t}{\tilde{x}}_{00}\right|+\left|c_{0}^{T}\int_{0}^{t}e^{A_{0}\left(t-\tau \right)}b_{0}u\left(\tau \right)d\tau \right|\\ \;+\left|{\tilde{c}}^{T}\int_{0}^{t}e^{\tilde{A}\left(t-\tau \right)}\tilde{b}u\left(\tau \right)d\tau \right|+\left(k_{1}+d\right)\left|u\left(t\right)\right|+k_{2}\left|y\left(t\right)\right|+k_{3}\left|y\left(t\right)u\left(t\right)\right| \end{array}$$

Since $\underset{0\leq t<\infty }{sup}\left|u\left(t\right)\right|\leq m_{u}<\frac{1-k_{2}}{k_{3}}<\infty$ and $A_{0}$ and $\tilde{A}$ are critically stable matrices then $\|e^{A_{0}t}\|\leq K_{0}e^{-\rho_{0}t}=K_{0}$ and $\|e^{\tilde{A}t}\|\leq \tilde{K}e^{-\tilde{\rho }t}=\tilde{K}$ for some real constants $K_{0}\geq 1$, $\tilde{K}\geq 1$, $\rho_{0}=\tilde{\rho }=0$ and; $\forall t\in R_{0+}$, and since $k_{2}+m_{u}k_{3}<1$, one gets the result after using twice in the above expression (15) the Cauchy-Schwarz inequality for integration of a product of real square-integrable functions $\int_{0}^{t}f\left(\tau \right)g\left(\tau \right)d\tau \leq {\left(\int_{0}^{t}f^{2}\left(\tau \right)d\tau \right)}^{1/2}{\left(\int_{0}^{t}g^{2}\left(\tau \right)d\tau \right)}^{1/2}$. Then, Property (i) follows directly. On the other hand, if G(s) and $\tilde{G}\left(s\right)$ are stable transfer functions then $A_{0}$ and $\tilde{A}$ are stability matrices, so that $\|e^{A_{0}t}\|\leq K_{0}e^{-\rho_{0}t}$ and $\|e^{\tilde{A}t}\|\leq \tilde{K}e^{-\tilde{\rho }t}$ for some real constants $K_{0}\geq 1$, $\tilde{K}\geq 1$, $\rho_{0}>0$ and $\tilde{\rho }>0$; $\forall t\in R_{0+}$. If, furthermore, the control input is uniformly bounded, but not necessarily square-integrable, subject to $\underset{0\leq t<\infty }{sup\left|u\left(t\right)\right|}\leq m_{u}<\frac{1-k_{2}}{k_{3}}$, one gets Property (ii) from (15). Also, if $d=k_{1}=k_{3}=0$, $k_{2}<1$ and $u\in L_{2}$ with $\int_{0}^{\infty }u^{2}\left(\tau \right)d\tau \leq M_{u}^{2}<\infty$, one gets the first part of Property (iii) by using the Cauchy-Schwartz inequality in the integrals of (15). Its last part follows directly by inspecting the limit superior when the input is jointly uniformly bounded and square-integrable. Finally, we have to prove Properties (iv)–(v). Note that if $u\in L_{2}$ then $\left|\left|y\right|-\left|\eta \right|\right|\in L_{2}$ from (2). Several cases can arise “a priori” for the square-integrability or not of y and η, namely, Case 1: $\eta \in L_{2}$, $y\in L_{2}$. The claim is proved. Case 2: $\eta \notin L_{2}$ then $y\notin L_{2}$, since otherwise $\left|\left|y\right|-\left|\eta \right|\right|\notin L_{2}$. Case 3: $y\notin L_{2}$, then $\eta \notin L_{2}$, since otherwise $\left|\left|y\right|-\left|\eta \right|\right|\notin L_{2}$. Cases 2 and 3 are similar in the sense that both $\eta ,y\notin L_{2}$ if both $\eta ,y\in L_{2}$. On the other hand, since $u\in L_{2}$ either u(t)→0 as t→∞ except, at most, on a finite or infinite set of isolated time instants of finite jump discontinuities $t_{j}\in {\left\{t_{k}\right\}}_{k\in Z_{0+}}$ or u(t)→0 as t→∞ except, at most, on a finite set of isolated time instants of Dirac jump discontinuities or a combination of both situations can arise. We now discuss the rebuttal of the above Case 2 by using contradiction arguments. It follows from (2) and Assumption A1 that:

$$\left|\left|y\left(t\right)\right|-\left|\eta \left(t\right)\right|\right|\geq \left(1-k_{2}-k_{3}\left|u\left(t\right)\right|\right)\left|y\left(t\right)\right|-k_{1}\left|u\left(t\right)\right|$$

Then, there is a sequence of ordered time instants ${\left\{t_{k}\right\}}_{k\in Z_{0+}}$ such that $t_{k+1}-t_{k}\geq T>0$; $\forall k\in Z_{0+}$ such that $u\left(t_{k}+\tau \right)\to 0$ as k→∞, and $1-k_{2}-k_{3}\left|u\left(t\right)\right|\geq 1-k_{2}-k_{3}\varepsilon_{k}>0$; for some ${\left\{\varepsilon_{k}\right\}}_{k\in Z_{0+}}\left(\subset R_{0+}\right)\to 0$ such that $\left|u\left(t\right)\right|\leq \varepsilon_{k}=\varepsilon \left(t_{k}\right)$; $\forall t\in {\left\{\left(t_{k},t_{k+1}\right)\right\}}_{k\in Z_{0+}}$, and:

$$\left|y\left(t\right)\right|\leq \frac{\left|\left|y\left(t\right)\right|-\left|\eta \left(t\right)\right|\right|+k_{1}\left|u\left(t\right)\right|}{1-k_{2}-k_{3}\left|u\left(t\right)\right|};\forall t\in {\left\{\left(t_{k},t_{k+1}\right)\right\}}_{k\in Z_{0+}}$$

In particular:

$$\left|y\left(t\right)\right|\leq \frac{\left|\left|y\left(t\right)\right|-\left|\eta \left(t\right)\right|\right|+k_{1}\left|u\left(t\right)\right|}{1-k_{2}-k_{3}\left|u\left(t\right)\right|};\;\forall t\in \left[t_{k},t_{k+1}\right),\;\forall k\in Z_{0+}$$

and, since $u\left(t_{k}+\tau \right)\to 0$ and $\left|\left|y\left(t_{k}+\tau \right)\right|-\left|\eta \left(t_{k}+\tau \right)\right|\right|+k_{1}\left|u\left(t_{k}+\tau \right)\right|\to 0$ for $\tau \in \left(t_{k},t_{k+1}\right)$ as k→∞, one has:

(16) $$\underset{\tau \in \left(t_{k},t_{k+1}\right),\;k\to \infty }{lim\;sup}\left(\left|y\left(t_{k}+\tau \right)\right|-\frac{\left|\left|y\left(t_{k}+\tau \right)\right|-\left|\eta \left(t_{k}+\tau \right)\right|\right|+k_{1}\;\left|u\left(t_{k}+\tau \right)\right|}{1-k_{2}-k_{3}\left|u\left(t_{k}+\tau \right)\right|}\right)\underset{\tau \in \left(t_{k},t_{k+1}\right),k\to \infty \left(\left|y\left(t_{k}+\tau \right)\right|\right)}{=lim\;sup}$$

so that there exists $\underset{\tau \in \left(t_{k},t_{k+1}\right),k\to \infty }{lim}y\left(t_{k}+\tau \right)=0$, and:

(17) $$\begin{array}{l} \int_{0}^{\infty }y^{2}\left(\tau \right)d\tau \leq 2\left(\int_{0}^{\infty }\frac{{\left|\left|y\left(\tau \right)\right|-\left|\eta \left(\tau \right)\right|\right|}^{2}}{{\left(1-k_{2}-k_{3}\left|u\left(\tau \right)\right|\right)}^{2}}d\tau +k_{1}^{2}\int_{0}^{\infty }\frac{u^{2}\left(\tau \right)}{{\left(1-k_{2}-k_{3}\left|u\left(\tau \right)\right|\right)}^{2}}d\tau \right)\\ \;\leq 2\left(\int_{0}^{t_{0}}\frac{{\left|\left|y\left(\tau \right)\right|-\left|\eta \left(\tau \right)\right|\right|}^{2}}{{\left(1-k_{2}-k_{3}\left|u\left(\tau \right)\right|\right)}^{2}}d\tau +k_{1}^{2}\int_{0}^{\infty }\frac{u^{2}\left(\tau \right)}{{\left(1-k_{2}-k_{3}\left|u\left(\tau \right)\right|\right)}^{2}}d\tau \right)\\ \;+\frac{2}{{\left(1-k_{2}-k_{3}\varepsilon_{0}\right)}^{2}}\left(\int_{t_{0}}^{\infty }{\left|\left|y\left(\tau \right)\right|-\left|\eta \left(\tau \right)\right|\right|}^{2}d\tau +k_{1}^{2}\int_{0}^{\infty }u^{2}\left(\tau \right)d\tau \right)\\ \;\leq 2M+2\left(M_{1}+M_{2}\right)=2\left[M+M_{1}+\frac{k_{1}^{2}M_{u}^{2}}{{\left(1-k_{2}-k_{3}\varepsilon_{0}\right)}^{2}}\right]<+\infty \end{array}$$

Since $t_{0}$ is finite, $\left|\left|y\right|-\left|\eta \right|\right|\in L_{2}$ and $u\in L_{2}$. Then, $y,\eta \in L_{2}$ which contradicts the assumption $y,\eta \notin L_{2}$ (Case 2). Thus, Case 2 (and thus Case 3) is impossible. Since G(s) is strictly stable and $u\in L_{2}$ then $\|x\|\in L_{2}$ for any finite $x_{0}$. Property (iv) has been proved. Now, if $u\in L_{\infty }\cap L_{2}$ with $\underset{0\leq t<\infty }{\|u_{\infty }\|=sup\left|u\left(t\right)\right|}\leq m_{u}<\frac{1-k_{2}}{k_{3}}$ then $\left|\left|y\right|-\left|\eta \right|\right|\in L_{\infty }\cap L_{2}$ from (2). The fact that $\left|y\right|,\left|\eta \right|\in L_{2}$ has been already proved in the proof of Property (iv) since $u\in L_{2}$ and $\left|y\right|,\left|\eta \right|\in L_{2}$. It has been also proved that $\underset{\tau \in \left(t_{k},t_{k+1}\right),k\to \infty }{lim}y\left(t_{k}+\tau \right)=0$. Thus, y(t) is bounded for $t\in \cup_{k\in Z_{0+}}\left(t_{k},\;t_{k+1}\right)$. It can be unbounded at time instants in [0, ∞) if there are impulsive controls and d>0 but, since $u\in L_{2}$, there is a connected time interval of infinite finite measure $\left[t_{a},\infty \right)\subset \cup_{k\in Z_{0+}}\left(t_{k},\;t_{k+1}\right)$ such that |y(t)|<+∞ for $t\in \left[t_{a},\infty \right)$, otherwise, $u\notin L_{2}$. From (2), $u\in L_{\infty }$ and $\bar{G}\in RH_{\infty }$, where $\bar{G}=G+\tilde{G}=G_{0}+\tilde{G}+d$, one gets also that ‖x(t)‖<+∞ for $t\in \left[t_{a},\infty \right)$.

Now, use the relationships $\|\bar{G}{f\|}_{2}\leq {\left\|\bar{G}\right\|}_{\infty }{\left\|f\right\|}_{2}$ if $f\in L_{2}$ and $\|\bar{G}f{\|}_{\infty }\leq {\left\|\bar{G}\right\|}_{\infty }\|f{\|}_{\infty }$ if $f\in L_{\infty }$, [23]. Thus, one has that $\eta \in L_{2}$ and $y\in L_{2}$ if $u\in L_{\infty }\cap L_{2}$ with $\|u{\|}_{\infty }=\underset{0\leq t<+\infty }{sup}\left|u\left(t\right)\right|\leq m_{u}<\frac{1-k_{2}}{k_{3}}$ and $\|u{\|}_{2}\leq M_{u}$ since $u\in L_{2}$ so that $\|y{\|}_{\infty }\leq \frac{m_{u}\left({\left\|\bar{G}\right\|}_{\infty }+k_{1}\right)}{1-k_{2}-k_{3}m_{u}}+K_{1}$; $\|y{\|}_{2}\leq \frac{\left({\left\|\bar{G}\right\|}_{\infty }+k_{1}\right)}{1-k_{2}-k_{3}m_{u}}M_{u}+K_{2}$ for some $0\leq K_{i}=K_{i}\left(x_{0}\right)<+\infty$; i=1,2.

Note that, if $y\in L_{\infty }$, $u\in L_{2}$ and $k_{2}<1$ so that $\eta \in L_{2}$ and $y\in L_{2}$ from Theorem 4(iv), one has following the proof guidelines of Theorem 4(v) that $\|y{\|}_{2}\leq \frac{\left({\left\|\bar{G}\right\|}_{\infty }+k_{1}+k_{3}\|y{\|}_{\infty }\right)}{1-k_{2}}M_{u}+K_{3}$ for some $0\leq K_{3}=K_{3}\left(x_{0}\right)<+\infty$. □

It is of interest to consider Theorem 4 jointly with the relevant previous non-negativity results of the input-output energy to get new combined results concerning the simultaneous non-negativity and boundedness of the input-output energy for certain time intervals of nonzero-measure. In this context, one has the following further results:

**Theorem** **5.** *Let Assumptions A1, A3 and A5 hold. In addition*, assume that one of the subsequent constraints hold:

*(1)*  Assumption A4 holds.
*(2)*  $k_{2}\leq 1$*and, furthermore,*$u\in L_{\infty }$*with*u(t)→0*as*t→∞.

*Then,*$0\leq E_{f}\left(t_{0},t\right)<+\infty$*;*$\forall t\left(\geq t_{0}\right)\in R_{0+}$*and any*$t_{0}\in R_{0+}$*. If*$t_{1}\left(>t_{0}\right)\in R_{+}$*and*u(t)*is nonzero for some time subinterval of*$\left[t_{0},t_{1}\right]$*of nonzero measure then*$0<E_{f}\left(t_{1},t\right)<+\infty$*;*$\forall t\in \left[t_{1},\infty \right)$.

**Proof.**  It follows directly from Corollary 1, Theorem 3 and Theorem 4(ii). □

Note that assumption A6 guarantees that Assumption A5 holds and that $u\in PC\cap L_{2}$ implies that $u\in L_{\infty }$ with u(t)→0 as t→∞. The whole input-output energy is non-negative and bounded for any time interval under Theorem 4 provided that the system is externally positive.

**Corollary** **4.** *Assume that the assumptions of Theorem 5 hold and, in addition, a state-space realization*R=(A,b,c,d)*of*$G\left(s\right)+\tilde{G}\left(s\right)$*is externally positive (or, alternatively, a state-space realization of*$G\left(s\right)+\tilde{G}\left(s\right)-d$*is externally positive and*d≥0*), and*$\eta :R_{0+}\times R_{0+}\times \left[0,t\right)\to R_{0+}$*subject to a control input*$u:R_{0+}\to R_{0+}$*and a initial state*$x_{0}\underline{≻}0$*. Then,*$0\leq E\left(t_{0},t\right)<+\infty$*;*$\forall t_{0},t\left(\geq t_{0}\right)\in R_{0+}$*and any*$t_{0}\in R_{0+}$.

**Proof.**  It follows directly from Theorem 5 and Corollary 2 and Theorem 4(ii) since $0\leq E_{u}\left(t_{0},t\right)<+\infty$ and $0\leq E_{f}\left(t_{0},t\right)<+\infty$; $\forall t_{0,}t\left(\geq t_{0}\right)\in R_{0+}$. □

**Remark** **8.** *Note that the non-negativity or, respectively, positivity results of the input-output energy of this section within positive time intervals are got based on the non-negativity or positivity of the integrands defining such an energy measure. Therefore, the input-output energy per time unity, that is the supplied power, is also non-negative or, respectively, positive in the corresponding results. If such an input-output energy (or, equivalently, the instantaneous supplied power) is non-negative for all time then the system is said to be hyperstable. In the most general case that such amounts are larger than some finite negative real constant independent of time, then the system is said to be passive [9,10,11,12]. Note from the above concepts that a hypestable system is also a passive system.*

## 3. Hyperstability and Asymptotic Hyperstability of the Closed-Loop System

The non-negativity positivity property of the input-output energy measure is now re-addressed under a feedback law of the form (3)–(4). If the jointly non-negativity/passivity is kept for the whole controllers satisfying (3)–(4) the closed-loop system, is said to be hyperstable. Consider the nonlinear time-varying differential system (1) under any controller (3)–(4) belonging to the class $\Phi_{0*}$. We will follow a classical nomenclature based in that used in [9] as follows:

(a) If $E_{z.s}\left(0,t\right)\geq 0$; $\forall t\in R_{0+}$ (i.e., the input-output energy measure of the uncontrolled system is non-negative for all time) then the feed-forward part of the system is said to be hyperstable. See, for example, Theorem 1. Note that such a condition $E_{z.s.}\left(0,t\right)\geq 0$; $\forall t\in R_{0+}$ which is guaranteed under several of the given results in Section 2, is a passivity condition of the feed-forward device of the control system if $u\in L_{2}$, [9,10,11].
  This condition holds, in particular, if the feed-forward system is linear (i.e., if η≡0) and $G\left(s\right)=G_{0}\left(s\right)+\tilde{G}\left(s\right)+d$ is {PR} what implies, furthermore, that it is stable as a result. It suffices that $G_{0}\left(s\right)\in \left\{PR\right\}$ and that $\tilde{G}\left(s\right)$ be strictly stable (which can be relaxed to $\tilde{G}\left(s\right)$ to be non-strictly stable with only a single critical pole with associated positive residual if $G_{0}\left(s\right)$ is strictly stable) with sufficiently small resonance peak related to the input-output interconnection gain d>0.
(b) If, in addition, $E_{z.s.}\left(0,t\right)>0$; $\forall t\in R_{+}$ (i.e., the input-output energy measure of the uncontrolled system is positive for all positive time) then the feed-forward part of the system is said to be asymptotically hyperstable. Such a conditions holds, in particular, if the feed-forward system is linear (i.e., if η≡0) and $G\left(s\right)=G_{0}\left(s\right)+\tilde{G}\left(s\right)+d$ is {SSPR} what implies, furthermore, that it is strictly stable.
(c) The set of all the controllers (3)–(4) of class $\Phi_{0*}=\left\{\Phi_{0\gamma }:\gamma \in R_{0+}\right\}$ is said to be a hyperstable class of controllers, each individual one being passive since its input-output measure satisfies the Popov’s inequality $\int_{0}^{t}\phi \left(y\left(\tau \right),\tau \right)y\left(\tau \right)d\tau \geq -\gamma >-\infty ;\forall t\in R_{0+}$ since $\phi \in \Phi_{0*}$. Consider instead the class of asymptotically hyperstable controllers $\Phi_{+*}=\left\{\Phi_{+\gamma }:\gamma \in R_{+}\right\}$, each one being (strictly) passive [10]. Note that if $u\in \Phi_{0*}$ then $u\in \Phi_{+*}$. Therefore, $\Phi_{0*}⊇\Phi_{+*}$. That is, the class of the hyperstable controllers includes that of the asymptotically hyperstable ones. However, the converse is not true. That is, $\Phi_{0*}\subset \Phi_{+*}$ does not hold with proper set inclusion.
(d) Hyperstability (respectively, asymptotic hyperstability) of a closed-loop dynamic system are properties of global (respectively, global asymptotic) stability under any member $\phi \in \Phi_{0*}$ of the hyperstable (respectively, asymptotically hyperstable $\phi \in \Phi_{+*}$) class of controllers, [5,6,10,20].

Note that if $k_{3}\neq 0$ then the control effort has to be saturated according to Assumption A4 for the validity of many of the given results. This fact needs to re-formulate the Popov’s inequality (3) [19] with another further constraint, which can in fact to be given equivalently in terms on a bounding inequality of the input-output energy of the feed-forward block, by taking into account the negative feedback action in the control effort. In this context, the following simple considerations are useful:

**Remarks** **9.** ***1*** *. If* $k_{3}=0$ *then (4) implies that* $\Phi_{0*}=\cup_{\gamma \in R_{0+}}\Phi_{0\gamma }$ *, where:*

(18) $$\Phi_{0\gamma }=\left\{\phi :R_{0+}\times R_{0+}\to R\;|\int_{0}^{t}u\left(\tau \right)y\left(\tau \right)d\tau \leq \gamma <\infty ;\forall t\in R_{0+}\right\};\;\gamma \in R_{0+}$$

*In the same way,*$\Phi_{+*}=\cup_{\gamma \in R_{+}}\Phi_{+\gamma }$, where:

(19) $$\Phi_{+\gamma }=\left\{\phi :R_{0+}\times R_{0+}\to R\;|\int_{0}^{t}u\left(\tau \right)y\left(\tau \right)d\tau \leq \gamma <\infty ;\forall t\in R_{0+}\right\};\;\gamma \in R_{+}$$

***2**. On the other hand, if*$k_{3}\neq 0$*and Assumption A4 is requested to hold then*$\Phi_{0*}$and$\Phi_{+*}$are, respectively, restricted to$\Phi_{r0*}\subset \Phi_{0*}\cap L_{\infty }$*and*$\Phi_{r+*}\subset \Phi_{+*}\cap L_{\infty }$*, where*$\Phi_{r0*}=\cup_{\gamma \in R_{0+}}\Phi_{r0\gamma }$*and*$\Phi_{r+*}=\cup_{\gamma \in R_{+}}\Phi_{r+\gamma }$ with:

(20) $$\Phi_{r0\gamma }=\{\phi :R_{0+}\times R_{0+}\to R\;|[(\int_{0}^{t}u\left(\tau \right)y\left(\tau \right)d\tau \leq \gamma <\infty )\wedge \;\left(\underset{0\leq \tau \leq t}{sup}\left|u\left(\tau \right)\right|\leq \frac{1-k_{2}}{k_{3}}\right);\;\forall t\in R_{0+}]\};\;\gamma \in R_{0+}$$

(21) $$\Phi_{r+\gamma }=\{\phi :R_{0+}\times R_{0+}\to R\;|[\left(\int_{0}^{t}u\left(\tau \right)y\left(\tau \right)d\tau \leq \gamma <\infty \right)\wedge \;\left(\underset{0\leq \tau \leq t}{sup}\left|u\left(\tau \right)\right|\leq \frac{1-k_{2}}{k_{3}}\right);\;\forall t\in R_{0+}]\};\;\gamma \in R_{+}$$

***3**. For getting conditions of asymptotic convergence to zero of the various control, output and state signals describing the dynamic system, the basic condition on the input square-integrability, and its eventual finite saturation, can require extra supporting constraints on its bounded time-derivative or its everywhere piece-wise continuity as addressed in the subsequent result.*

***4**. Note that Assumption A5 is stronger than Assumption A2 while Assumption A5 guarantees Assumption A2.*

**Theorem** **6.** *The following properties hold:*

***(i)***  *If Assumptions A1–A4 hold and*$\phi \in \Phi_{0*}$then$0\leq E_{f}\left(0,t\right)\leq \gamma <+\infty$*;*$\forall t\in R_{0+}$.
***(ii)***  *If Assumptions A1–A3 hold,*$k_{3}=0$*(so that Assumption A4 is superfluous) and*$\phi \in \Phi_{0*}$thenu(t)*is everywhere zero except possibly on a subinterval of*$R_{0+}$*of zero measure. Also,*$u\in L_{2}$*,*$\underset{0\leq t<\infty }{esssup}\left|u\left(t\right)\right|<+\infty$*,*$\underset{t_{0}\leq t<\infty }{sup}\left|u\left(t\right)\right|<+\infty$*for some finite*$t_{0}\in R_{0+}$*,*u(t)→0*as*t→∞*except possibly on a set of zero measure of isolated bounded jump discontinuities,*$x\in L_{\infty }$*and*$y:\left[t_{0},\infty \right)$*for any finite initial sate*$x_{0}\in R^{n}$*so that the closed-loop system is hyperstable for the class*$\Phi_{0*}$ of controllers.
  *If*$k_{3}\neq 0$*and Assumption A4 holds then*$x\in L_{\infty }$*and*$y\in L_{\infty }$*if*$k_{2}+k_{3}{\bar{M}}_{u}<1$*where*${\bar{M}}_{u}=(max\;\underset{0\leq t<\infty }{sup\;\left|u\left(t\right)\right|},M_{u})$.
***(iii)***  *If, Assumptions A1, A3 hold, Assumption A2 is modified to its stronger version of Assumption A5 and* $\phi \in \Phi_{+*}$ *holds then* $0<E_{f}\left(0,t\right)\leq \gamma <+\infty$ *;* $\forall t\in R_{+}$ *,* $u\in L_{2}$ *,* $\underset{0\leq t<\infty }{esssup}\left|u\left(t\right)\right|<+\infty$ *,* $\underset{t_{0}\leq t<\infty }{sup}\left|u\left(t\right)\right|<+\infty$ *for some finite* $t_{0}\in R_{0+}$ *,* u(t)→0 *as* t→∞ *, except possibly on a set of zero measure of isolated bounded jump discontinuities,* $x\in L_{2}^{n}\cap L_{\infty }^{n}$ *and* $y,\eta ,\left(y-\eta \right)\in L_{2}\cap L_{\infty }$ *,* x(t)→0 *as* t→∞ *, and* y(t)→0 *as* t→∞ *and* η(t)→0 *as* t→∞ *, except possibly on a set of zero measure of isolated bounded jump discontinuities, for any given finite initial sate* $x_{0}\in R^{n}$ *. As a result, the closed-loop system is asymptotically hyperstable for the class of controllers* $\Phi_{+*}$ *.*
***(iv)***  *If Assumptions A1–A4 hold and* $\phi \in \Phi_{r0*}\cap L_{\infty a}$ *with* $k_{3}\neq 0$ *then* $u\in L_{2}\cap L_{\infty a}$ *,* $\underset{0\leq t<\infty }{sup}\left|u\left(t\right)\right|\leq \frac{1-k_{2}}{k_{3}}<+\infty$ *,* u(t)→0 *as* t→∞ *,* $x\in L_{\infty }^{n}$ *and* $y\in L_{\infty }$ *for any finite initial sate* $x_{0}\in R^{n}$ *so that the closed-loop system is hyperstable for the class* $\Phi_{r0*}\cap L_{\infty a}$ *of controllers.*
***(v)***  *If Assumptions A1, A3, A4 and A5 hold and* $\phi \in \Phi_{r+*}\cap L_{\infty a}$ *then* $0<E_{f}\left(0,t\right)\leq \gamma <+\infty$ *;* $\forall t\in R_{+}$ *,* $\underset{0\leq t<\infty }{sup}\left|u\left(t\right)\right|\leq \frac{1-k_{2}}{k_{3}}<+\infty$ *,* u(t)→0 *as* t→∞ *,* $x\in L_{2}^{n}\cap L_{\infty }^{n}$ *and* $L_{\infty }^{n}$ *,* x(t)→0 *as* t→∞ *and* y(t)→0 *as* t→∞ *and* η(t)→0 *as* t→∞ *, for any given finite initial sate* $x_{0}\in R^{n}$ *so that the closed-loop system is asymptotically hyperstable for the class* $\Phi_{r+*}\cap L_{\infty a}$ *of controllers.*

**Proof.**  Property (i) is direct from the negative feedback control (3)-(4), Theorem 1 and Remark 9.1. From Property (i), $0\leq E_{f}\left(0,t\right)\leq \gamma <+\infty$ and from Theorem 1, $u\in L_{2}$ if $\phi \in \Phi_{0*}$. Since $\int_{0}^{t}u^{2}\left(\tau \right)d\tau \leq M_{u}<+\infty$ and u(t) is bounded except possibly on a set $S_{zm}\subset R_{0+}$ of zero measure. Two cases can arise if this assertion in untrue, namely: Case a) there is at least one finite escape time $\left[t_{0},\;t_{0}+\varepsilon_{0}\right)$ for some $t_{0}\in R_{0+}$ and $\varepsilon_{0}\in R_{+}$ so that $\left|u\left(t\right)\right|\geq m_{0u}$; $\forall t\in \left[t_{0},\;t_{0}+\varepsilon_{0}\right)$ and $m_{u}$ being arbitrarily large. Thus, $\varepsilon m_{0u}^{2}\leq \int_{0}^{t}u^{2}\left(\tau \right)d\tau \leq M_{u}^{2}<+\infty$ so that $m_{0u}\leq \frac{M_{u}}{\sqrt{\varepsilon }}<+\infty$, a contradiction and a finite escape time cannot occur on an interval of nonzero measure. Case b) u(t) has infinitely many impulses (i.e., jumps of infinity amplitudes at certain time instants) at an infinite sequence of time instants ${\left\{t_{i}\right\}}_{i\in Z_{0+}}\subset R_{0+}$ of amplitudes $K_{i}\delta \left(t-t_{i}\right)$, $K_{i}\in R\backslash \left\{0\right\}$ and δ(t) denoting the Dirac distribution. It follows that $+\infty =\underset{n\to \infty }{lim}\sum_{i=0}^{n}K_{i}^{2}\leq \underset{t\to \infty }{lim\;sup}\int_{0}^{t}u^{2}\left(\tau \right)d\tau \leq M_{u}^{2}<+\infty$, a contradiction, so the left-hand side should have necessarily a finite number of additive terms. Then, there is some finite $t_{0}\in R_{0+}$ such that u(t) is impulse -free for $t\geq t_{0}$. As a consequent result to these two impossible cases, one concludes that $\underset{0\leq t<\infty }{esssup}\left|u\left(t\right)\right|<+\infty$, $\underset{t_{0}\leq t<\infty }{sup}\left|u\left(t\right)\right|<+\infty$ for some finite $t_{0}\in R_{0+}$. Note that a third possible Case c related to u(t) having finitely many or infinitely many finite discontinuity jumps on a set of isolated time instants (i.e., a set zero measure) is compatible with its proved boundedness properties. Another consequence of the above discussion is that, u(t)→0 as t→∞, except possibly on a set of isolated time instants, since $\underset{0\leq t<\infty }{esssup}\left|u\left(t\right)\right|<+\infty$, $\underset{t_{0}\leq t<\infty }{sup}\left|u\left(t\right)\right|<+\infty$ and the input can posses only finite discontinuity jumps on isolated time instants (Case c). The facts that:

(1) $x\in L_{\infty }$ (since eventual input Dirac impulses at isolated time instants generate jump unbounded discontinuities in $\dot{x}\left(.\right)$ but translate in bounded discontinuities in x(.)) and,
(2) $y:\left[t_{0},\infty \right)$ is bounded (since $d\underset{t_{0}\leq t<\infty }{sup}\left|u\left(t\right)\right|<+\infty$) for any finite initial sate $x_{0}\in R^{n}$ follow directly from the fact that $G_{0}\left(s\right)+\tilde{G}\left(s\right)+d$ is a stable transfer function. If, in addition, $k_{3}\neq 0$ and Assumption A4 holds then the control input is uniformly bounded so that $x\in L_{\infty }$ and $y\in L_{\infty }$ if $k_{2}+k_{3}{\bar{M}}_{u}<1$ where ${\bar{M}}_{u}=(max\;\underset{0\leq t<\infty }{sup\left|u\left(t\right)\right|},M_{u})$ (see Theorem 4(iii)). Property (ii) has been proved.

To prove Property (ii), note under the given Assumptions A1, A3 and A5, one gets from Corollary 1 and each control function $\phi \in \Phi_{+*}$ that:

(22) $$0<E_{f}\left(0,t\right)\leq \gamma <+\infty ;\;\forall t\in R_{+}$$

for some $\gamma \in R_{+}$, eventually depending on $\phi \in \Phi_{\gamma }$, for any used $\phi \in \Phi_{+*}$. From Assumption A5, $\bar{G}\left(s\right)=G\left(s\right)+d=G_{0}\left(s\right)+\tilde{G}\left(s\right)+d$ is strictly stable since it is strictly positive real and it is also strongly strictly positive real with zero relative degree since the input-output interconnection gain of the feed-forward linear block d is positive according to Assumption A3. Thus, $\underset{\omega \in R_{0+}}{min}Re\;\left(\bar{G}\left(i\omega \right)\right)=\underset{\omega \in R}{min}Re\;\left(G_{0}\left(i\omega \right)+\tilde{G}\left(i\omega \right)+d\right)>0$. From Corollary 1 and Theorem 1, one has that $u\in L_{2}$ implying also that u(t)→0 almost everywhere as t→∞ (it can have finite jump discontinuities on a set of zero measure and a finite number of Dirac impulsive discontinuities on a finite time interval $\left[0,t_{0}\right)$ of $R_{0+}$). Thus, $\underset{0\leq t<\infty }{esssup}\left|u\left(t\right)\right|<+\infty$, $\underset{t_{0}\leq t<\infty }{sup}\left|u\left(t\right)\right|<+\infty x\in L_{2}^{n}\cap L_{\infty }^{n}$ (note that $\bar{G}\in RH_{\infty }$ since it is in {SPR}) and $y,\eta ,\left(y-\eta \right)\in L_{2}\cap L_{\infty }$, x(t)→0 as t→∞, and y(t)→0 as t→∞ and η(t)→0 as t→∞, except possibly on a set of zero measure of isolated bounded jump discontinuities, for any given finite initial sate $x_{0}\in R^{n}$. Property (iii) has been proved.

Property (iv) can be proved under close arguments as those used for the proof of Property (iii). However, the asymptotic properties of square-integrability and convergence to zero of the state and output signals are not guaranteed for any finite initial conditions, since $G\notin RH_{\infty }$ while it is only guaranteed to be critically stable and positive real. Note that Assumption A4 implies a boundedness condition on the input only if $k_{3}\neq 0$. However, it does not imply such a boundedness if $k_{3}=0$. Since $k_{2}\leq 1$, if $k_{3}>0$, then $u\in L_{2}\cap L_{\infty }$ (subject to the supremum constrained from Assumption A4) and if $k_{3}=0$ then $u\in L_{2}$. The proof of Property (v) is similar to that of Property (iii). Since the input is everywhere bounded for all time from Assumption A4, the boundedness and asymptotic convergence to zero of the state and output signals hold without excluding eventually sets of zero measure of time instants. □

A direct weaker result than Theorem 5(iv) due to restriction on the admissible functions in the Popov’s inequality to be satisfied by the controller is the following one:

**Corollary** **5.** *If Assumptions A1, A3 and A5 hold and* $\phi \in \Phi_{+*}\cap \left(PC_{a}\cup \left(C\cap \left(\dot{\phi }\in L_{\infty }\right)\right)\right)$ *then,* $0<E_{f}\left(0,t\right)\leq \gamma <+\infty$ *;* $\forall t\in R_{+}$ *,* $u\in L_{2}$ *,* $\underset{0\leq t<\infty }{sup}\left|u\left(t\right)\right|<+\infty$ *,* $\underset{0\leq t<\infty }{sup}\left|u\left(t\right)\right|<+\infty$ *,* u(t)→0 *as* t→∞ *,* $x\in L_{2}^{n}\cap L_{\infty }^{n}$ *,* $y\in L_{2}\cap L_{\infty }$ *,* x(t)→0 *as* t→∞ *and* y(t)→0 *as* t→∞ *for any given finite initial sate* $x_{0}\in R^{n}$ *so that the closed-loop system is asymptotically hyperstable for the class* $\Phi_{+*}\cap \left(PC_{a}\cup \left(C\cap \left(\dot{\phi }\in L_{\infty }\right)\right)\right)$ *of controllers, [20].*

**Outline** **of** **Proof.** It is based on the property that if $\phi \in \Phi_{+*}$ and $u\in L_{2}$ (following Theorem 6 (iii)) then u(t)→0 as t→∞ provided that ϕ is piece-wise continuous except on a set of zero measure of bounded discontinuities (so that it can not be unbounded since it is square-integrable and has to vanish asymptotically since it is in $L_{2}$) or provided that ϕ is continuous and everywhere time-differentiable with time-derivative possibly subject to bounded discontinuities. □

## 4. Examples on Epidemic Models Subject to Vaccination and Treatment Controls

Epidemic modelling has been shown to be a relevant tool in Public Health prevention and infectious diseases treatment. The existing background literature is abundant not only at a mathematical modelling level but at the related application studies and practical cases as well. An important investigation tool for that is the design of vaccination and treatment controls. See, for instance, [26,27,28,29,30,31] and references therein. It is well- known that vaccination has made a very relevant contribution to global health [32,33,34,35]. In particular, two major infectious diseases, namely, polio and rinderpest have been eradicated, [35]. On the other hand, the global coverage of vaccination against certain infectious diseases, like measles or polio, has led to them to be targeted for eradication or almost eradicated, respectively. It is pointed out in [34] that the focus has to be addressed towards the following objectives: (a) the support to the design and more efficiently delivery of new vaccines; (b) the acceleration of the practical implementation of designed vaccines; and (c) the implementation of policies and mechanisms to make the vaccination achievable to those who need them, [34]. Our objective in those applications examples is linked to the third above goal in the sense that we give analytical design tools for vaccination controls which are based on feedback information on the subpopulations of the epidemic models. It is not the main interest the biological nature or such vaccines but really how to implement then on the susceptible population. It turns out that the vaccination control might be only effective when applied to susceptible (i.e., not yet infected) population. In parallel, there are also (for instance, either antibacterial or antiviral) potential treatment controls which can be effective when applied on the already infected population. The design of mechanism to regulate the administration of such controls is of interest in epidemic models to the light of the above theoretical development. Note that the asymptotic hyperstability of the incremental linearized system around the disease-free equilibrium point allows the stabilization of disturbances of the solution trajectory caused by infection escaping from the equilibrium point under a very wide class of vaccination/treatment controls. This arises from the very essential property of the hyperstability consisting of global stability under a very wide class of nonlinear feedback controllers. The hyperstability concept relies on the stabilization with any member of a very wide class of controllers satisfying a Popov’s inequality, not just with some particular controller. Therefore, the controller gains are not necessarily constant but time-varying and, in general, non-linear. This fact is emphasized for the designed clases of controllers of the examples. See, for instance, Assumptions A7 to A9 of the Example 1 displayed below.

Two related application examples are now given to illustrate the results of the above sections:

**Example** **1.** *Consider the subsequent SIR epidemic model subject to vaccination and treatment controls:*

$$\dot{S}\left(t\right)=-\beta S\left(t\right)I\left(t\right)-u_{V}\left(t\right)$$

(23) $$\dot{I}\left(t\right)=\beta S\left(t\right)I\left(t\right)-\gamma I\left(t\right)-u_{a}\left(t\right)$$

$$\dot{R}\left(t\right)=\gamma I\left(t\right)+\lambda_{V}\left(t\right)u_{V}\left(t\right)+\lambda_{a}\left(t\right)u_{a}\left(t\right)$$

*subject to initial non-negative conditions, that is, min(S(0),I(0),R(0))≥0, where S(t), I(t) and R(t) are the susceptible, infective and immune (or recovered) subpopulations, respectively, and $u_{V}\left(t\right)$ and $u_{a}\left(t\right)$ are the vaccination and treatment controls on the susceptible and infective, respectively. In the above model, β is the infective parameter and γ is the removal parameter giving the rates at which the infective individuals become immune. Finally, $\lambda_{V}\left(t\right)\in \left[0,\;1\right]$ and $\lambda_{a}\left(t\right)\in \left[0,\;1\right]$ are the functions characterizing lost of efficiency of the respective controls if they are less than unity. The interpretation can be that they are due to abrupt allergic reaction causing slight mortality if they are close to unity or in other situations if simply the imperfect vaccination and treatment could generate another subpopulation in the model but the particular concerns are not of interest for our study objectives in this paper. By analysis convenience, define the following auxiliary function which includes the quadratic nonlinear term:*

(24) $$V\left(t\right)=-\beta S\left(t\right)I\left(t\right)-u_{V}\left(t\right)$$

*then the model can be, equivalently, rewritten as follows:*

$$\dot{S}\left(t\right)=-V\left(t\right)$$

(25) $$\dot{I}\left(t\right)=-\gamma I\left(t\right)-V\left(t\right)-u_{V}\left(t\right)-u_{a}\left(t\right)$$

$$\dot{R}\left(t\right)=\gamma I\left(t\right)+\lambda_{V}\left(t\right)u_{V}\left(t\right)+\lambda_{a}\left(t\right)u_{a}\left(t\right)$$

*Now, define proportionality gains $\rho_{V}\left(t\right)$, $\rho_{u_{V}}\left(t\right)$ and $\rho_{u_{a}}\left(t\right)$ to link V(t), and the vaccination and treatment controls $u_{V}\left(t\right)$ and $u_{a}\left(t\right)$ to a primary scalar control u(t) as follows:*

(26) $$V\left(t\right)=\rho_{V}\left(t\right)u\left(t\right);\;u_{V}\left(t\right)=\rho_{uv}\left(t\right)u\left(t\right);\;u_{a}\left(t\right)=\rho_{ua}\left(t\right)u\left(t\right)$$

*The above constraints lead to:*

(27) $$\rho_{V}\left(t\right)u\left(t\right)=-\beta S\left(t\right)I\left(t\right)-\rho_{uv}\left(t\right)u\left(t\right)$$

*so that:*

(28) $$-\left(V\left(t\right)+u_{V}\left(t\right)+u_{a}\left(t\right)\right)=-\rho_{I}\left(t\right)u\left(t\right);\;\lambda_{V}\left(t\right)u_{V}\left(t\right)+\lambda_{a}\left(t\right)u_{a}\left(t\right)=\rho_{R}\left(t\right)u\left(t\right)$$

*where:*

(29) $$\rho_{I}\left(t\right)=-\left(\rho_{V}\left(t\right)+\rho_{uv}\left(t\right)+\rho_{ua}\left(t\right)\right);\;\rho_{R}\left(t\right)=\lambda_{V}\left(t\right)\rho_{uv}\left(t\right)+\lambda_{a}\left(t\right)\rho_{ua}\left(t\right)$$

(30) $$u\left(t\right)=-\frac{\beta S\left(t\right)I\left(t\right)}{\rho_{V}\left(t\right)+\rho_{uv}\left(t\right)}$$

*Thus, the model driven by the primary control u(t) becomes equivalently:*

$$\dot{S}\left(t\right)=-\rho_{V}\left(t\right)u\left(t\right)$$

(31) $$\dot{I}\left(t\right)=-\gamma I\left(t\right)-\rho_{I}\left(t\right)u\left(t\right)$$

$$\dot{R}\left(t\right)=\gamma I\left(t\right)+\rho_{R}\left(t\right)u\left(t\right)$$

*Define the state vector as $x\left(t\right)={\left(S\left(t\right),I\left(t\right),R\left(t\right)\right)}^{T}$, the scalar measurable output y(t) is defined later on for different situations and consider the following further assumptions:*

*Assumptions*

**A7.***The efficiency controls lost*$\lambda_{V}\left(t\right)=\lambda_{V}$*and*$\lambda_{a}\left(t\right)=\lambda_{a}$*and the proportionality gains*$\rho_{V}\left(t\right)=\rho_{V}$*,*$\rho_{I}\left(t\right)=\rho_{I}$*and*$\rho_{R}\left(t\right)=\rho_{R}$*;*$\forall t\in R_{0+}$ are constant.
**A8.**$\rho_{uv}\left(t\right)\leq -\rho_{V}$*and*$\rho_{ua}\left(t\right)\leq -\rho_{I}$*;*$\forall t\in R_{0+}$*implying that*$\rho_{uv}\left(t\right)=-\left(\rho_{I}+\rho_{V}+\rho_{ua}\left(t\right)\right)<-\rho_{V}$*;*$\forall t\in R_{0+}$.
**A9.***The primary control is generated via nonlinear/time-varying feedback as*$u\left(t\right)=-\phi \left(y\left(t\right),\;t\right)\leq \gamma_{0}\left(t\right)/y\left(t\right)$*, with*$\phi \in \Phi_{+*}$is generated by output feedback satisfies a Popov’s-type inequality such that$0<\gamma \left(t\right)=\int_{0}^{t}\gamma_{0}\left(\tau \right)d\tau \leq \bar{\gamma }<+\infty$*;*$\forall t\in R_{+}$*for some integrable*$\gamma_{0}:R_{0+}\to R$*implying that*$\int_{0}^{t}y\left(\tau \right)u\left(t\right)d\tau \leq \bar{\gamma }$*;*$\forall t\in R_{+}$*, and*$0>\rho_{V}+\rho_{uv}\left(t\right)\geq -\frac{\beta S\left(t\right)I\left(t\right)y\left(t\right)}{\gamma_{0}\left(t\right)}$*;*$\forall t\in R_{0+}$*, equivalently,*
  (32) $$\gamma_{0}\left(t\right)\geq \frac{\beta S\left(t\right)I\left(t\right)}{\left|\rho_{V}+\rho_{uv}\left(t\right)\right|}$$
  *and*$\gamma_{0}\left(t\right)$*is integrable on*[0, ∞)*so that*S(t)I(t)*is also integrable on*[0, ∞).

*Then, the closed-loop system becomes:*

(33) $$\dot{x}\left(t\right)=Ax\left(t\right)+bu\left(t\right)$$

*under non-negative initial components, with:*

(34) $$A=\left[\begin{array}{ccc} 0 & 0 & 0\\ 0 & -\gamma & 0\\ 0 & \gamma & 0 \end{array}\right];\;b=\left[\begin{array}{c} -\rho_{V}\\ -\rho_{I}\\ \rho_{R} \end{array}\right]$$

*Case 1: Assume that the measurable output y(t) is the susceptible subpopulation S(t) so that $c=e_{1}={\left(1,\;0,0\right)}^{T}$. Then,*

$$e_{1}^{T}{\left(sI-A\right)}^{-1}b=\left(1,0,0\right){\left[\begin{array}{ccc} s & 0 & 0\\ 0 & s+\gamma & 0\\ 0 & -\gamma & s \end{array}\right]}^{-1}\left[\begin{array}{c} -\rho_{V}\\ -\rho_{I}\\ \rho_{R} \end{array}\right]=\frac{1}{s^{2}\left(s+\gamma \right)}\left[s\left(s+\gamma \right)\;,0,0\right]\left[\begin{array}{c} -\rho_{V}\\ -\rho_{I}\\ \rho_{R} \end{array}\right]=-\frac{\rho_{V}}{s}$$

*Case 2: Assume that the measurable output y(t) is the infective subpopulation I(t) so that $c=e_{2}={\left(0,\;1,\;0\right)}^{T}$. Then,*

$$e_{2}^{T}{\left(sI-A\right)}^{-1}b=\left(0,1,0\right){\left[\begin{array}{ccc} s & 0 & 0\\ 0 & s+\gamma & 0\\ 0 & -\gamma & s \end{array}\right]}^{-1}\left[\begin{array}{c} -\rho_{V}\\ -\rho_{I}\\ \rho_{R} \end{array}\right]=\frac{1}{s^{2}\left(s+\gamma \right)}\left[0,s^{2},0\right]\left[\begin{array}{c} -\rho_{V}\\ -\rho_{I}\\ \rho_{R} \end{array}\right]=-\frac{\rho_{I}}{s+\gamma }$$

*Case 3: Assume that the measurable output y(t) is the recovered subpopulation R(t) so that $c=e_{3}={\left(0,\;0,\;1\right)}^{T}$. Then,*

$$e_{3}^{T}{\left(sI-A\right)}^{-1}b=\left(0,0,1\right){\left[\begin{array}{ccc} s & 0 & 0\\ 0 & s+\gamma & 0\\ 0 & -\gamma & s \end{array}\right]}^{-1}\left[\begin{array}{c} -\rho_{V}\\ -\rho_{I}\\ \rho_{R} \end{array}\right]=\frac{1}{s^{2}\left(s+\gamma \right)}\left[0,0,s\left(s+\gamma \right)\right]\left[\begin{array}{c} -\rho_{V}\\ -\rho_{I}\\ \rho_{R} \end{array}\right]=\frac{\rho_{R}}{s}$$

Note that the transfer function of Case 1 (y(t)=S(t)) is positive real if $\rho_{V}<0$, that of Case 2 (y(t)=S(t)) is positive real if $\rho_{I}<0$ and that of Case 3 is positive real if $\rho_{R}>0$. None of the three transfer functions is strictly positive real under a primary control satisfying Assumption A9. If the output is taken as any linear combination of the three subpopulations the resulting transfer function of the linear feed-forwards part is not strictly positive real either. Thus, global asymptotic stability (i.e., asymptotic hyperstability) cannot be concluded for controls $\phi \in \Phi_{+*}$ under Assumption A9 but only hyperstability.

**Example** **2.** *Let us now consider instead the following linearized epidemic model of dimension two whose incremental state is*$\tilde{x}\left(t\right)={\left(\tilde{S}\left(t\right),\tilde{R}\left(t\right)\right)}^{T}$*, related to the disease-free equilibrium point*$x_{e}={\left(S_{e},\;R_{e}\right)}^{T}$*, which includes linear proportional to the susceptible vaccination efforts reinforced by a nonlinear eventually time-varying incremental term*$\tilde{u}=-\phi$*,*$\phi \in \Phi_{+*}$, which uses feedback information from the defined incremental outputy(t)*which could be the incremental susceptible or the incremental immune or a linear combination of them:*

(35) $$\dot{\tilde{S}}\left(t\right)=-\left(\mu +\beta +k_{1}\right)\tilde{S}\left(t\right)-\tilde{u}\left(t\right)=\left(\mu +\beta \right)\tilde{S}\left(t\right)-\left(k_{1}\tilde{S}\left(t\right)+\tilde{u}\left(t\right)\right)$$

(36) $$\dot{\hat{R}}\left(t\right)=\left(1-\alpha \right)\beta \tilde{S}\left(t\right)-\mu \tilde{R}\left(t\right)+\left(k_{1}\tilde{S}\left(t\right)+\tilde{u}\left(t\right)\right)$$

*under finite non-negative initial given conditions. In this linearized incremental model, the infection is assumed to be instantaneous,*μ*is the natural mortality,*β*is the infection rate per individual per unity of time,*0≤α≤1*is the infected mortality, and its complementary fraction*(1−α)*characterizes the rates or infected survivors which are transferred directly from the susceptible subpopulation to the recovered one. The parameter*$k_{1}$*is the rate of susceptible in individuals which are vaccinated by proportional feedback linear control, so they are removed from the susceptible dynamics and transferred to the recovered one, and*$\tilde{u}\left(t\right)=-\phi \left(t\right)$*;*$\forall t\in R_{+}$*with*$\phi \in \Phi_{+*}$is a complementary term added to the above vaccination effort. In this way, the whole incremental feedback vaccination control in (35) and (36) is given by$\tilde{V}\left(t\right)=k_{1}\tilde{S}\left(t\right)+\tilde{u}\left(t\right)$*;*$\forall t\in R_{+}$. *The particular case of the above model for the incremental vaccination-free case, i.e.,*$\tilde{V}\left(t\right)\equiv 0$*is described in [26] following the pioneering infection transmission models proposed by Daniel Bernouilli (1700–1782) following a smallpox disease which widespread along Europe while affecting a large proportion of the population and causing around 10% of the mortality of minors. The survivors were found to be immune to further attack but left scarred for life. The estimations of Bernoulli’s studies are described in detail in [26], were it is also pointed out that, in 1760, Bernouilli read his paper “Essai d´une nouvelle analyse de la mortalité causée par la petite vérole et des advantages de l´inoculation pour la prévenir” to the French Royal Academy of Sciences in Paris. Note that the idea of “inoculation” is simply to inject attenuated live virus obtained from patients with mild case of smallpox (“variolation” [26]). The above model is inspired in that proposed by Bernouilli for cohorts of individuals born in a particular year and with an age-specific per capita death rate. The state equation can be compactly written as follows:*

(37) $$\dot{\tilde{x}}\left(t\right)=A\tilde{x}\left(t\right)+b\tilde{u}\left(t\right)=\left[\begin{array}{cc} -\left(\mu +\beta +k_{1}\right) & 0\\ \left(1-\alpha \right)\beta +k_{1} & -\mu \end{array}\right]\tilde{x}\left(t\right)+\left[\begin{array}{c} -1\\ 1 \end{array}\right]\tilde{u}\left(t\right)$$

*Note that the matrix of dynamics is a stability matrix with eigenvalues*$-\left(\mu +\beta +k_{1}\right)$*and*−μ. Then:

(38) $${\left(sI-A\right)}^{-1}b=\frac{1}{\left(s+\mu \right)\left(s+\mu +\beta +k_{1}\right)}\left[\begin{array}{cc} s+\mu & 0\\ -\left(\left(1-\alpha \right)\beta +k_{1}\right) & s+\mu +\beta +k_{1} \end{array}\right]\left[\begin{array}{c} -1\\ 1 \end{array}\right]\;=\left[\begin{array}{c} -\frac{1}{s+\mu +\beta +k_{1}}\\ \frac{1}{s+\mu }+\frac{\left(1-\alpha \right)\beta +k_{1}}{\left(s+\mu \right)\left(s+\mu +\beta +k_{1}\right)} \end{array}\right]$$

Several cases are of interest, namely:

(a) if $\tilde{u}\equiv 0$ then $\tilde{x}\left(t\right)\to 0$ exponentially as t→∞ since A is a stability matrix.

(b) Assume that $\tilde{y}\left(t\right)=\tilde{S}\left(t\right)+d\tilde{u}\left(t\right)$. Then the transfer function from $\tilde{u}\left(t\right)$ to $\tilde{y}\left(t\right)$ is $G_{\tilde{S}}\left(s\right)=d-\frac{1}{s+\mu +\beta +k_{1}}=d\frac{s+\left(\mu +\beta +k_{1}\right)-1/d}{s+\mu +\beta +k_{1}}$. It is strongly strictly positive real if $d>d_{c}=1/\left(\mu +\beta +k_{1}\right)$. Note that the critical interconnection gain $d_{c}=d_{c}\left(k_{1}\right)$ is strictly decreasing with $k_{1}$ for given constant β and μ and $d_{c}=d_{c}\left(\beta \right)$ is strictly decreasing with β for given constants μ and $k_{1}$. It follows that $u\left(t\right)=-\phi \left(\tilde{y}\left(t\right)\right)\to 0$ as t→∞ for any real function $\phi :R_{0+}\to R_{0+}$ which satisfies Popov’s inequality. In particular, one can choose an incremental control generated by a functional $\phi \left(\tilde{y}\left(t\right)\right)$ which satisfies $\tilde{u}\left(t\right)=-\phi \left(\tilde{y}\left(t\right)\right)\leq \frac{\gamma_{0}\left(t\right)}{\tilde{S}\left(t\right)+\tilde{u}\left(t\right)}$ such that $\gamma_{0}:R_{0+}\to R_{0+}$ is any integrable function on [0,∞), in particular, any non-negative function of exponential negative order so exponentially vanishing. Defining the convex parabola $P\left(\tilde{u}\left(t\right)\right)={\tilde{u}}^{2}\left(t\right)+\tilde{S}\left(t\right)\tilde{u}\left(t\right)-\gamma_{0}\left(t\right)$, the admissible controls leading to asymptotic hyperstability are those which fulfil $\tilde{u}\left(t\right)\in \left[{\tilde{u}}_{1}\left(t\right),\;{\tilde{u}}_{2}\left(t\right)\right]$ with:

$${\tilde{u}}_{1}\left(t\right)=\frac{-\tilde{S}\left(t\right)-\sqrt{{\tilde{S}}^{2}\left(t\right)+4\gamma_{0}\left(t\right)}}{2};\;{\tilde{u}}_{2}\left(t\right)=\frac{-\tilde{S}\left(t\right)+\sqrt{{\tilde{S}}^{2}\left(t\right)+4\gamma_{0}\left(t\right)}}{2}$$

As a result, the total incremental vaccination effort takes the form $\tilde{V}\left(t\right)\in \left[{\tilde{V}}_{1}\left(t\right),\;{\tilde{V}}_{2}\left(t\right)\right]$ with:

$${\tilde{V}}_{1}\left(t\right)=\left(k_{1}-1/2\right)\tilde{S}\left(t\right)-\frac{\sqrt{{\tilde{S}}^{2}\left(t\right)+4\gamma_{0}\left(t\right)}}{2};\;{\tilde{V}}_{2}\left(t\right)=\left(k_{1}-1/2\right)\tilde{S}\left(t\right)+\frac{\sqrt{{\tilde{S}}^{2}\left(t\right)+4\gamma_{0}\left(t\right)}}{2}$$

It follows that the incremental control, output and state variables related to the disease-free equilibrium point exponentially vanish, i.e., $\tilde{u}\left(t\right)\to 0$, $\tilde{x}\left(t\right)\to 0$, $\tilde{y}\left(t\right)\to 0$ as t→∞. Note that along the transient and because of the structure (37) and the positivity of the product $\tilde{y}\left(t\right)\tilde{u}\left(t\right)$, one concludes that if $\tilde{u}\left(t\right)>0$ (in practice, close to the upper-bound ${\tilde{u}}_{2}\left(t\right)$), then $\tilde{y}\left(t\right)=\tilde{S}\left(t\right)+d\tilde{u}\left(t\right)\geq 0$, then $\tilde{S}\left(t\right)\geq -d\tilde{u}\left(t\right)\geq -d{\tilde{u}}_{2}\left(t\right)$, so the incremental susceptible subpopulation could be negative along the transient which can be of interest to reduce the incremental susceptibility. However, if $\tilde{u}\left(t\right)<0$ then $\tilde{y}\left(t\right)=\tilde{S}\left(t\right)-d\left|\tilde{u}\left(t\right)\right|\leq 0$ so that $\tilde{S}\left(t\right)\geq d\left|\tilde{u}\left(t\right)\right|$ concluding the incremental susceptibility is increased along the transient. As a result, it seems of interest to choose the incremental control with positive values being close to its admissible upper- bound. The incremental recovered subpopulation is positive along the transient in both cases in view of (37).

(c) Assume that $\tilde{y}\left(t\right)=\tilde{R}\left(t\right)+d\tilde{u}\left(t\right)$. Then the transfer function from $\tilde{u}\left(t\right)$ to $\tilde{y}\left(t\right)$ is $G_{R}\left(s\right)=d+\frac{1}{s+\mu }+\frac{\left(1-\alpha \right)\beta +k_{1}}{\left(s+\mu \right)\left(s+\mu +\beta +k_{1}\right)}=d+\frac{s+\mu +\beta +k_{1}+\left(1-\alpha \right)\beta +k_{1}}{\left(s+\mu \right)\left(s+\mu +\beta +k_{1}\right)}$, which strongly strictly positive real if d>0. Thus, the appropriate incremental control which guarantees asymptotic hyperstability is $\tilde{u}\left(t\right)=-\phi \left(\tilde{y}\left(t\right)\right)\leq \frac{\gamma_{0}\left(t\right)}{\tilde{R}\left(t\right)+\tilde{u}\left(t\right)}$ with $\gamma_{0}\left(t\right)$ being non-negative and integrable on [0, ∞). Following a similar reasoning to that of the above case, one concludes that $\tilde{u}\left(t\right)\in \left[{\tilde{u}}_{3}\left(t\right),\;{\tilde{u}}_{4}\left(t\right)\right]$ and that the admissible incremental vaccination effort is $\tilde{V}\left(t\right)\in \left[{\tilde{V}}_{3}\left(t\right),\;{\tilde{V}}_{4}\left(t\right)\right]$ with:

$${\tilde{u}}_{3}\left(t\right)=\frac{-\tilde{R}\left(t\right)-\sqrt{{\tilde{R}}^{2}\left(t\right)+4\gamma_{0}\left(t\right)}}{2};\;{\tilde{u}}_{4}\left(t\right)=\frac{-\tilde{R}\left(t\right)+\sqrt{{\tilde{R}}^{2}\left(t\right)+4\gamma_{0}\left(t\right)}}{2}$$

$${\tilde{V}}_{3}\left(t\right)=\left(k_{1}-1/2\right)\tilde{R}\left(t\right)-\frac{\sqrt{{\tilde{R}}^{2}\left(t\right)+4\gamma_{0}\left(t\right)}}{2};\;{\tilde{V}}_{4}\left(t\right)=\left(k_{1}-1/2\right)\tilde{R}\left(t\right)+\frac{\sqrt{{\tilde{R}}^{2}\left(t\right)+4\gamma_{0}\left(t\right)}}{2}$$

It is seen from (37) that $\tilde{u}\left(t\right)>0$ is of interest along the transient in order to decrease the incremental susceptibility and to increase the incremental recovered subpopulation.

## 5. Conclusions

This paper has developed a formalism for hyperstability and asymptotic hyperstability of controlled dynamic systems whose feed-forward part excluding potential controls consist of the additive contributions of a known linear dynamics, an unknown one and unknown nonlinear disturbances under wide classes of controllers which satisfy a Popov’s-type inequality. The known linear part is given by a positive real transfer function, the unknown dynamics is assumed stable but it is unknown except some “a priori” knowledge of its resonance peak, that is, the maximum gain in the frequency domain. The nonlinear contribution to the dynamics is not known precisely, but is known its growing rule depending on input, output and the input-output product through time in the sense that available upper- bounds of the linear weighting factors for those values are known. The structure of the controlled systems generalizes the usual one stated as a basis for hyperstable designs by incorporating linear and nonlinear uncertainties. A robust asymptotic hyperstable property of the closed-loop system is proved to be achieved under a set of constraints and the design of any member of classes of controllers satisfying Popov’s-type inequalities. These kinds of controllers are not very restrictive compared to the usual single ones which achieve global asymptotic Lyapunov’s hyperstability. In fact, all the classes of asymptotic hyperstable controllers satisfy a certain Lyapunov function for the whole class. On the other hand, it is emphasized the interest of such designs in the field of vaccination and treatment feedback controls of epidemic models since it is well-known the relevance of those controls in Public Health Management. In particular, those control designs are of a major interest for the acceleration of the practical implementation of vaccination policies and mechanisms to make the vaccination achievable to those who really need them. Two classical epidemic models under those class of controllers have been discussed in the paper. The objective is that the vaccination and treatment controllers have a designed component which is designed “ad hoc” under hyperstability tools for the automatic generation of a fast control action against any potential deviation of the disease-free equilibrium point due to variations in either the equilibrium population numbers (which indicates a disease regrowth), in the parameterization or in the known modelled dynamics (which indicates presumably either a change on the disease defining parameters or a dynamics disturbance contribution, due for instance, to interchange of populations with other neighboring environments). The contributions of the paper consist of: (1) the extension of the classical hyperstability theory to the presence of unmodeled, or not very precisely parameterized linear and nonlinear contributions to the dynamics, (2) the application of the obtained results to epidemic models under vaccination and treatment controls under a wide class of asymptotically hyperstable controllers, and (3) the use of those control designs to rapidly fight against the deviations of the steady disease- free equilibrium point due to those kinds disturbances or unmodeled dynamics and/or changes in the equilibrium population levels. In the future, it is claimed to extend the results to the hyperstability znd asymptotic of the whole nonlinear epidemic models rather than to the incremental models around the disease- free equilibrium points. 
